# An Updated Phylogenetic Assessment and Taxonomic Revision of *Perenniporia* sensu lato (Polyporales, Basidiomycota)

**DOI:** 10.3390/jof9020173

**Published:** 2023-01-28

**Authors:** Xing Ji, Yi-Fei Sun, Dong-Mei Wu, Neng Gao, Bao-Kai Cui

**Affiliations:** 1 Institute of Microbiology, School of Ecology and Nature Conservation, Beijing Forestry University, Beijing 100083, China; 2 Xinjiang Academy of Agricultural and Reclamation Sciences/Xinjiang Production and Construction Group Key Laboratory of Crop Germplasm Enhancement and Gene Resources Utilization, Biotechnology Research Institute, Shihezi 832000, China

**Keywords:** taxonomy, multigene phylogeny, polypore, systematics

## Abstract

*Perenniporia* is an important genus of Polyporaceae. In its common acceptation, however, the genus is polyphyletic. In this study, phylogenetic analyses on a set of *Perenniporia* species and related genera were carried out using DNA sequences of multiple loci, including the internal transcribed spacer (ITS) regions, the large subunit nuclear ribosomal RNA gene (nLSU), the small subunit mitochondrial rRNA gene (mtSSU), the translation elongation factor 1-α gene (TEF1) and the b-tubulin gene (TBB1). Based on morphology and phylogeny, 15 new genera, viz., *Aurantioporia*, *Citrinoporia*, *Cystidioporia*, *Dendroporia*, *Luteoperenniporia*, *Macroporia*, *Macrosporia*, *Minoporus*, *Neoporia*, *Niveoporia*, *Rhizoperenniporia*, *Tropicoporia*, *Truncatoporia*, *Vanderbyliella*, and *Xanthoperenniporia*, are proposed; 2 new species, *Luteoperenniporia australiensis* and *Niveoporia subrusseimarginata*, are described; and 37 new combinations are proposed. Illustrated descriptions of the new species are provided. Identification keys to *Perenniporia* and its related genera and keys to the species of these genera are provided.

## 1. Introduction

*Perenniporia* Murrill, typified by *P. medulla-panis* (Jacq.) Donk, is a large and cosmopolitan genus. The genus has been redefined by Decock and Stalpers [1], who designated a neotype. Species in this genus have annual to perennial, resupinate to pileate basidiocarps, a dimitic to trimitic hyphal system with clamped generative hyphae, cyanophilous and variably dextrinoid skeletal hyphae, and thick-walled, ellipsoid to subglobose, truncate or not, cyanophilous, and variably dextrinoid basidiospores [2,3].

In the last few decades, *Perenniporia* has been intensively studied and the number of species has increased significantly. Many new species were published based on morphological characters [1,4,5,6,7,8,9,10,11,12,13,14] or based on morphological characters and phylogenetic analysis [15,16,17,18,19,20,21,22,23,24,25,26,27,28,29].

These multiple additions considerably enlarged the morphological concept of *Perenniporia*. A recent trend is to make the morphologically distinct species or morphologically homogeneous alliance separate from *Perenniporia*. For instance, *Perenniporiella* Decock & Ryvarden [30] was separated from *Perenniporia* based on non-truncate basidiospores. This was later confirmed by Robledo et al. [31] by phylogenetic data. *Hornodermoporus*, *Truncospora*, and *Vanderbylia* are usually treated as synonyms of *Perenniporia* [2,32,33,34], but have been shown by phylogenetic studies to also represent clades distinct from the *Perenniporia* s. s. clade, each morphologically homogenous [3,17,19,20,26,31].

The current phylogenetic analysis confirmed that *Perenniporia* is polyphyletic, and some monophyletic clades have been separated from *Perenniporia* s. l. and recognized as independent genera [3,35,36,37]. Based on such characters as the acyanophily and amyloidity of skeletal hyphae dissolving in KOH, as well as the non-dextrinoidity of basidiospores and the phylogenetic data from the rDNA sequences of ITS and nLSU, *P. narymica* (Pilát) Pouzar was segregated into *Yuchengia* B.K. Cui & K.T. Steffen [35]. Wu et al. [36] also separated *Perenniporia minutissima* (Yasuda) T. Hatt. & Ryvarden from *Perenniporia* as *Perenniporiopsis minutissima* (Yasuda) C.L. Zhao based on the phylogenetic analyses, as well as the morphological differences of rigidly osseous basidiocarps when there are dry and large basidiospores. Cui et al. [3] used multiple genes of ITS, nLSU, nSSU, mtSSU, TEF1, TBB1, RPB1, and RPB2 and proposed the separation of *Perenniporia hattorii* Y.C. Dai and B.K. Cui from *Perenniporia* into *Amylosporia* B.K. Cui, C.L. Zhao & Y.C. Dai because of its amyloid skeletal hyphae and basidiospores, and *P. subadusta* (Z.S. Bi & G.Y. Zheng) Y.C. Dai from *Perenniporia* into *Murinicarpus* B.K. Cui & Y.C. Dai on account of its stipitate basidiocarps and cystidia in the hymenium. Chen et al. [37] pointed out that *Perenniporia subacida* (Peck) Donk formed a well-supported lineage that is distinct from the *Perenniporia* s. s. clade and proposed that *P. subacida* should be treated in a new genus named *Poriella* C.L. Zhao.

Although some monophyletic lineages have been assigned a new genus name (for example, *Amylosporia*, *Murinicarpus*, *Perenniporiopsis*, *Poriella*, and *Yuchengia* [3,35,36,37]), the positions of other *Perenniporia* species are still doubtful, and some species should be separated from *Perenniporia*. In order to clarify the taxonomy and phylogeny of *Perenniporia* s. l., reliable specimens and sequences were studied using morphological methods and phylogenetic analyses of ITS, nLSU, mtSSU, TEF1, and TBB1.

## 2. Materials and Methods

### 2.1. Morphological Studies

The specimens examined in this study are deposited in the herbaria of the Institute of Microbiology, Beijing Forestry University, China (BJFC) and the Institute of Applied Ecology, Chinese Academy of Sciences, China (IFP). Macro-morphological descriptions were based on field notes. Special color terms followed the Petersen protocol [38]. Micro-morphological data were obtained from dried specimens and observed under a light microscope according to Sun et al. [39] and Liu et al. [40]. Sections were studied at a magnification up to 1000× using a Nikon Eclipse 80i microscope and phase contrast illumination (Nikon, Tokyo). Drawings were made with the aid of a drawing tube. Microscopic features, measurements, and drawings were made from slide preparations stained with Cotton Blue and Melzer’s reagent. Spores were measured from sections cut from the tubes. To represent variation in the size of spores. Five percent of measurements were excluded from each end of the range and given in parentheses. The following abbreviations are used: IKI = Melzer’s reagent, IKI− = non-dextrinoid and inamyloid, KOH = 5% potassium hydroxide, CB = Cotton Blue, CB+ = cyanophilous, L = mean spore length (arithmetic average of all spores), W = mean spore width (arithmetic average of all spores), Q = variation in the L/W ratios between the specimens studied, and n = number of spores (a) measured from given number (b) of specimens.

### 2.2. DNA Extraction, Amplification and Sequencing

A CTAB rapid plant genome extraction kit-DN14 (Aidlab Biotechnologies Co., Ltd., Beijing, China) was used to extract total genomic DNA from dried specimens and to perform the polymerase chain reaction (PCR) according to the manufacturer’s instructions, with some modifications [3,41]. The ITS regions were amplified with the primer pairs ITS5 and ITS4 [42]. The nLSU regions were amplified with the primer pairs LR0R and LR7 (http://www.biology.duke.edu/fungi/mycolab/primers.htm, accessed on 12 October 2021). The mtSSU regions were amplified with the primer pairs MS1 and MS2 [42]. Part of TEF1 was amplified with the primer pairs EF1-983F and EF1-1567R [43]. TBB1 was amplified with the primer pairs Bt-1a and Bt-1b [44]. The PCR procedures for different DNA sequences were the same as those used by Cui et al. [3]. The PCR products were purified and sequenced at the Beijing Genomics Institute (BGI), China, with the same primers. All newly generated sequences and additional sequences downloaded from GenBank are listed in Table 1.

### 2.3. Phylogenetic Analyses

*Heterobasidion annosum* (Fr.) Bref. and *Stereum hirsutum* (Willd.) Pers. were used as outgroups for the ITS + nLSU analysis [45], *Fomitopsis pinicola* (Sw.) P. Karst. and *Daedalea quercina* (L.) Pers. were selected as outgroups for the ITS + nLSU + mtSSU + TEF1 + TBB1 analysis. All sequences were aligned in MAFFT 7 [46] (http://mafft.cbrc.jp/alignment/server/, accessed on 11 June 2022) and manually adjusted in BioEdit [47]. Maximum Likelihood (ML) and Bayesian Inference (BI) phylogenetic analyses were performed as described by Sun et al. [48]. ML studies and Bayesian inference were applied to the ITS + nLSU and ITS + nLSU + mtSSU + TEF1 + TBB1 datasets.

ML studies were conducted with RAxML-HPC through the Cipres Science Gateway (https://www.phylo.org, accessed on 12 June 2022) and involved 1000 ML searches under the GTRGAMMA model. Only the maximum likelihood best tree from all searches was kept. In addition, 1000 rapid bootstrap replicates were run with the GTRCAT model to assess the reliability of the nodes.

BI was performed with MrBayes v3.1.2 (Ronquist and Huelsenbeck, Sweden) [49]. Four Markov chains were run from random starting trees for 6 million generations for ITS + nLSU and for 13 million generations for ITS + nLSU + mtSSU + TFF1 + TBB1, and trees were sampled every 100th generation. The first 25% of trees were discarded as burn-in, and the remaining trees were used to calculate Bayesian posterior probabilities (BPP) of the clades.

Trees were viewed in FigTree v1.4.2 (http://tree.bio.ed.ac.uk/software/figtree/, accessed on 12 June 2022). Branches that received bootstrap support for maximum likelihood (BS) and Bayesian posterior probabilities (BPP) ≥ 50% (BS) and ≥0.90 (BPP) were considered significantly supported. The final concatenated sequence alignment and the retrieved topology were deposited in TreeBase (http://purl.org/phylo/treebase, accessed on 29 November 2022; submission ID: 29932).

## 3. Results

### 3.1. Phylogeny

**Figure 1:**
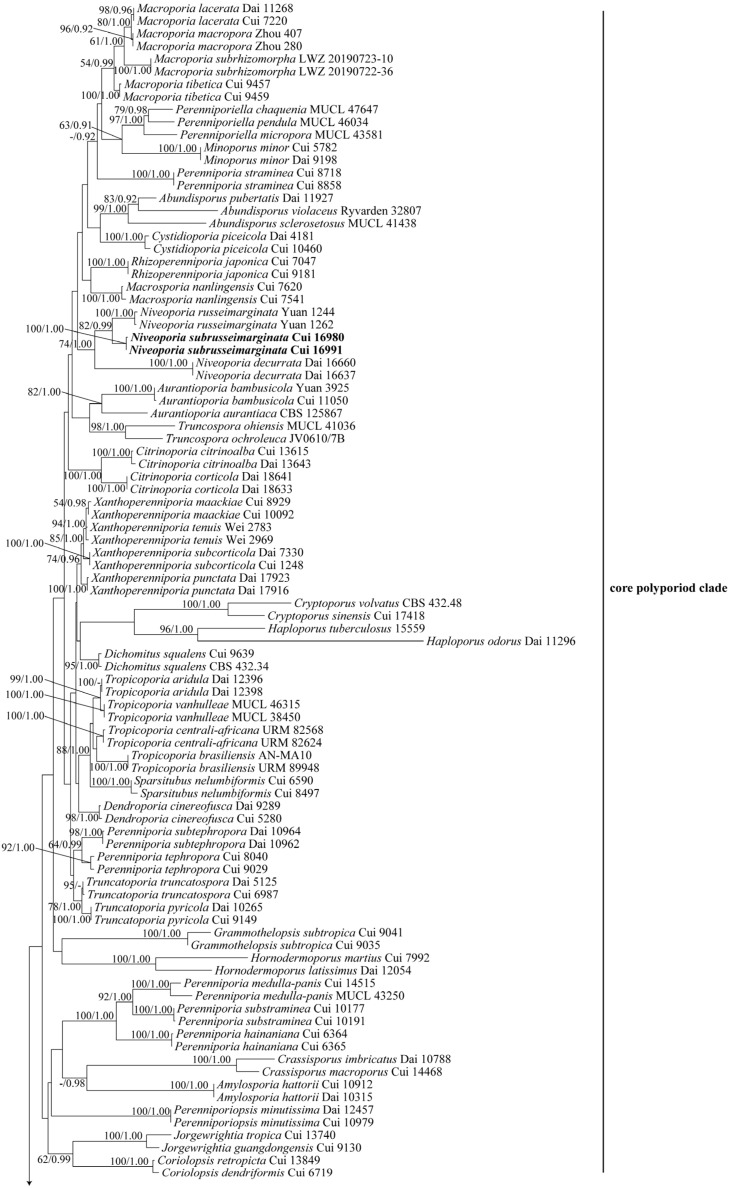

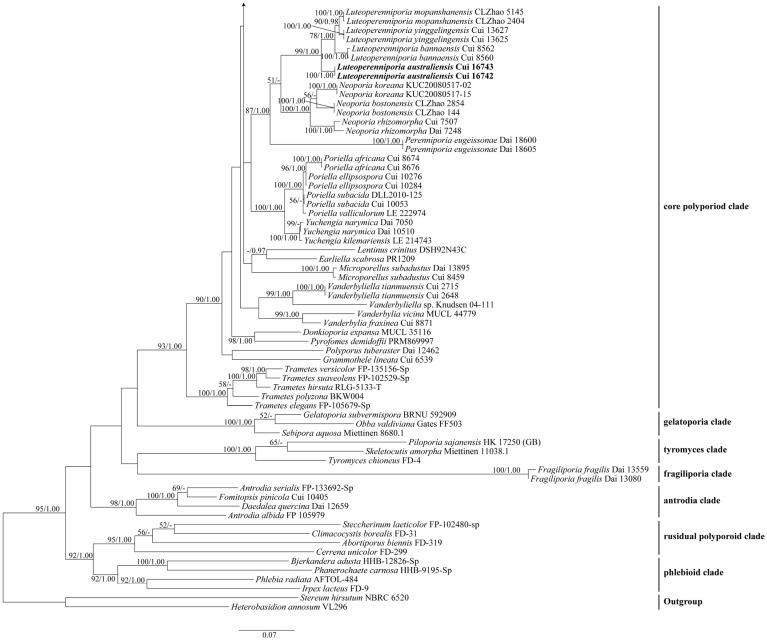
Maximum likelihood tree illustrating the phylogeny of *Perenniporia* and related species in Polyporales based on the ITS + nLSU dataset. Branches are labeled with maximum likelihood bootstrap support values (≥50%) and Bayesian posterior probabilities (≥0.90).

The combined ITS + nLSU dataset included sequences from 159 fungal samples representing 108 taxa. The dataset had an aligned length of 2215 characters, of which 1257 characters were constant, 235 were variable and parsimony-uninformative, and 723 were parsimony-informative. BI analysis generated topologies similar to those of ML analysis, with an average standard deviation of split frequencies at 0.004614 (BI). The topology from the ML analysis with a maximum likelihood bootstrap (BS) ≥ 50% and Bayesian posterior probabilities (BPP) ≥ 0.90 labeled on branches is shown (Figure 1).

**Figure 2:**
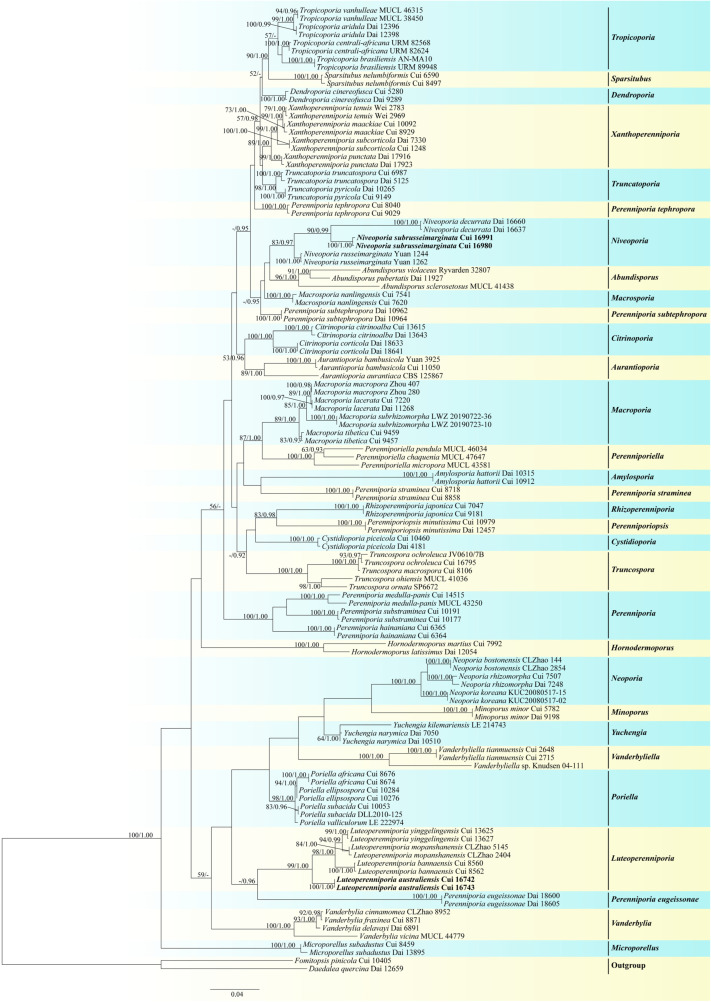
Maximum likelihood tree illustrating the phylogeny of *Perenniporia* and related genera based on the ITS + nLSU + mtSSU + TEF1 + TBB1 dataset. Branches are labeled with maximum likelihood bootstrap support values (≥50%) and Bayesian posterior probabilities (≥0.90).

The combined five-gene (ITS, nLSU, mtSSU, TEF1, TBB1) sequence dataset included sequences from 119 fungal samples representing 70 taxa. The dataset had an aligned length of 3498 characters, of which 2285 characters were constant, 226 were variable and parsimony-uninformative, and 987 were parsimony-informative. BI analysis generated topologies similar to those of ML analysis, with an average standard deviation of split frequencies = 0.007253 (BI). The topology from the ML analysis with a maximum likelihood bootstrap (BS) ≥ 50% and Bayesian posterior probabilities (BPP) ≥ 0.90 labeled on branches is shown (Figure 2).

### 3.2. Taxonomy

***Aurantioporia*** B.K. Cui & Xing Ji, gen. nov.

MycoBank: MB 847338

Differs from other genera by its resupinate, rhizomorphic basidiocarps with an orange pore surface, a dimitic hyphal system with arboriform skeletal hyphae, tissues becoming violet in KOH, ellipsoid, truncate, and slightly dextrinoid basidiospores.

Type species: *Aurantioporia bambusicola* (Choeyklin, T. Hatt. & E.B.G. Jones) B.K. Cui & Xing Ji

Etymology: *Aurantioporia* (Lat.) refers to the orange pore surface of the genus.

Basidiocarps are annual to perennial and resupinate with rhizomorphs. The pore surface is yellow to orange when fresh, grayish orange, and orange-brown to dark orange when dry; pores are round to angular; dissepiments thin, entire. The subiculum is extremely thin and cream to pale orange. The tubes are concolorous with pore surface. The hyphal system is dimitic; generative hyphae with clamp connections; skeletal hyphae arboriform, IKI−, CB+; tissues become violet to dark in KOH. Basidiospores are ellipsoid, truncate, hyaline, thick-walled, smooth, slightly dextrinoid, and CB+.

Notes: In our study, *P. aurantiaca* (A. David & Rajchenb.) Decock & Ryvarden and *P. bambusicola* Choeyklin, T. Hatt. & E.B.G. Jones formed a single well-supported clade (82% BS, 1.00 BPP, Figure 1; 89% BS, 1.00 BPP, Figure 2), distant from the *Perenniporia* s. s. clade. Morphologically, the two species differ from *Perenniporia* s. s. species by the combination of a rhizomorphic basidiocarp with an orange pore surface, a dimitic hyphal system with arboriform skeletal hyphae, and tissues becoming violet in KOH. Therefore, *Aurantioporia* gen. nov. is proposed to include *Perenniporia aurantiaca* and *P. bambusicola*.

***Aurantioporia aurantiaca*** (A. David & Rajchenb.) B.K. Cui & Xing Ji, comb. nov.

MycoBank: MB 847362

Basionym: *Pyrofomes aurantiacus* A. David & Rajchenb., Mycotaxon 22(2): 312 (1985).

≡ *Perenniporia aurantiaca* (A. David & Rajchenb.) Decock & Ryvarden, Mycol. Res. 103(9): 1140 (1999).

For a detailed description of *Perenniporia aurantiaca*, see David and Rajchenberg [50] and Decock and Ryvarden [4].

Notes: *Aurantioporia aurantiaca* was originally described in *Pyrofomes* by David and Rajchenberg [50] and later was transferred to *Perenniporia* by Decock and Ryvarden [4]. The sequence of *Aurantioporia aurantiaca* from French Guyana (type locality) fell into the *Aurantioporia* clade in our phylogeny.

***Aurantioporia bambusicola*** (Choeyklin, T. Hatt. & E.B.G. Jones) B.K. Cui & Xing Ji, comb. nov.

MycoBank: MB 847363

Basionym: *Perenniporia bambusicola* Choeyklin, T. Hatt. & E.B.G. Jones, Fungal Diversity 36: 122 (2009).

For a detailed description of *Perenniporia bambusicola*, see Choeyklin et al. [51] and Cui et al. [3].

Notes: *Aurantioporia bambusicola* was first described in *Perenniporia* from Thailand [51]. It is characterized by resupinate basidiocarps with an orange pore surface, a dimitic hyphal system with non-dextrinoid skeletal hyphae, tissues becoming violet to dark in KOH, oblong-ellipsoid, and truncate basidiospores. This species only grows on bamboo. *Aurantioporia aurantiaca* also shares an orange pore surface, but *Aurantioporia aurantiaca* grows on hardwood trees [51].

Specimen examined: CHINA. Yunnan, Cangyuan County, Banlao, on bamboo, 11 July 2013, Cui 11050 (BJFC).

**Key to species of *Aurantioporia***

1. Growing on bamboo; distributed in Southeast Asia ………………………………………………………………………………………………… *A. bambusicola*
1. Growing on other hardwoods; distributed in neotropical areas………………………………………………………………………………………*A. aurantiaca*

***Citrinoporia*** B.K. Cui & Xing Ji, gen. nov.

MycoBank: MB 847346

Differs from other genera by its slightly cushion shape, yellow pore surface, a dimitic hyphal system with dextrinoid, and cyanophilous shortly arboriform vegetative hyphae and ellipsoid, truncate, thick-walled, dextrinoid, and cyanophilous basidiospores.

Type species:

Etymology: *Citrinoporia* (Lat.) refers to the yellowish pore surface of the genus.

Basidiocarps are annual to perennial and resupinate. The pore surface is white to yellow; pores are round. Subiculum is cream to buff, corky. Tubes are buff to pale brown and corky to hard corky. The hyphal system is dimitic: generative hyphae with clamp connections; skeletal hyphae arboriform, IKI−, CB+; tissues becoming pale brown to black in KOH. Cystidia is absent, cystidioles are present. Basidiospores are ellipsoid, truncate, hyaline, thick-walled, smooth, dextrinoid, and CB+.

Notes: In our ITS + nLSU and five-gene phylogenetic analyses, *P. citrinoalba* B.K. Cui, C.L. Zhao & Y.C. Dai and *P. corticola* (Corner) Decock clustered together and formed a clade distinct from the *Perenniporia* s. s clade with full support (100% BS, 1.00 BPP, Figure 1; 100% BS, 1.00 BPP, Figure 2). Morphologically, this clade differs from the *Perenniporia* s. s. by its yellow pore surface and dimitic hyphal system. Thus, the new genus is set up and these two new combinations are proposed. Based on five-gene phylogenetic analysis, *Citrinoporia* is sister to *Aurantioporia* as they have the same overall morphology, yellow pore surface, dimitic hyphal system, and ellipsoid, truncate basidiospores, but the former differs in absence of rhizomorphs.

***Citrinoporia citrinoalba*** (B.K. Cui, C.L. Zhao & Y.C. Dai) B.K. Cui & Xing Ji, comb. nov.

MycoBank: MB 847364

Basionym: *Perenniporia citrinoalba* B.K. Cui, C.L. Zhao & Y.C. Dai, Fungal Diversity 97: 270 (2019)

For a detailed description of *Perenniporia citrinoalba*, see Cui et al. [3].

Notes: *Citrinoporia citrinoalba* was newly described in *Perenniporia* from tropical China [2]. It is characterized by annual and resupinate basidiocarps with white to yellow pore surfaces, a dimitic hyphal system with dextrinoid and cyanophilous skeletal hyphae, tissues becoming pale brown to black in KOH, and broadly ellipsoid and truncate basidiospores.

Specimens examined: CHINA. Hainan, Qiongzhong County, Limushan Forest Park, on fallen trunk of Castanopsis, 15 June 2014, Dai 13643 (holotype, BJFC), on fallen angiosperm trunk, 18 November 2015, Cui 13615 (BJFC).

***Citrinoporia corticola*** (Corner) B.K. Cui & Xing Ji, comb. Nov.

MycoBank: MB 847365

Basionym: *Parmastomyces corticola* Corner, Beih. Nova Hedwigia 96: 96 (1989).

≡ *Perenniporia corticola* (Corner) Decock, Mycologia 93(4): 776 (2001).

= *Perenniporia dipterocarpicola* T. Hatt. & S.S. Lee, Mycologia 91(3): 525 (1999)

Notes: *Citrinoporia corticola* was originally described in *Parmastomyces* Kotl. & Pouzar from Malaysia by Corner [52] as having a monomitic hyphal system with simple-septate generative hyphae. Decock [53] studied the type specimens of *Parmastomyces corticola* and confirmed that this species has a dimitic hyphal system with clamped generative hyphae and transferred the species to *Perenniporia*. *Citrinoporia corticola* and *Citrinoporia citrinoalba* share yellow pore surfaces, dimitic hyphal structures, and truncate basidiospores, but the basidiospores of *C. citrinoalba* (5.5–6 × 4.7–5.2 µm) [3] are larger than those of *Citrinoporia corticola* (4.4–5 × 3.4–4 μm) [53].

Specimens examined: MALAYSIA. Selangor, Kota Damansara, Community Forest Reserve, on angiosperm stump, 17 April 2018 Dai 18633, 18641 (BJFC).

**Key to species of *Citrinoporia***

1. Basidiospores 4.4–5 μm; growing mainly on trees of Dipterocarpaceae……………………………………………………………………………… *C. corticola*
1. Basidiospores 5.5–6 µm; growing on trees of Fagaceae ……………………………………………………………………………………………… *C. citrinoalba*

***Cystidioporia*** B.K. Cui & Xing Ji, gen. nov.

MycoBank: MB 847348

Differs from other genera by its resupinate basidiocarps, slightly dextrinoid and cyanophilous skeletal hyphae, presence of thick-walled cystidia, and thick-walled, oblong-ellipsoid, truncate, slightly dextrinoid, and cyanophilous basidiospores.

Type species: *Cystidioporia piceicola* (Y.C. Dai) B.K. Cui & Xing Ji

Etymology: *Cystidioporia* (Lat.) refers to resembling *Perenniporia* but with cystidia.

Basidiocarps are annual to biennial, resupinate, soft corky when fresh, and hard corky when dry. Pore surface is cream to buff when fresh and pale yellowish upon drying. Pores are round and large; dissepiments are thin, entire. Subiculum is yellowish ochraceous and corky. Tubes are yellowish ochraceous or straw yellow and corky. Hyphal system is dimitic to trimitic; generative hyphae with clamp connections; skeletal hyphae is slightly dextrinoid, CB+; tissues unchanged in KOH. Cystidia present, thick-walled, strongly CB+. Basidiospores are ellipsoid, truncate, hyaline, thick-walled, smooth, slightly dextrinoid, and CB+.

Notes: In our present phylogenetic analyses (Figure 1 and Figure 2), two specimens of *Perenniporia piceicola* Y.C. Dai formed a single clade distant from the *Perenniporia* s. s. clade. Moreover, this species has thick-walled cystidia, large pores, and basidiospores (pores 2–3 per mm, basidiospores 11–14 × 5.4–7.5 µm) [8] which are different from other species of *Perenniporia*. Thus, the new genus is set up, and the following combination is proposed.

***Cystidioporia piceicola*** (Y.C. Dai) B.K. Cui & Xing Ji, comb. nov.

MycoBank: MB 847366

Basionym: *Perenniporia piceicola* Y.C. Dai, Ann. Bot. Fenn. 39(3): 173 (2002).

For a detailed description of *Perenniporia piceicola*, see Dai et al. [8].

Notes: *Cystidioporia piceicola* was originally described in *Perenniporia* by Dai et al. [8]; it is characterized by resupinate basidiocarps, thick-walled cystidia, large pores and basidiospores, and usually grows on *Picea* and *Abies*.

Specimens examined: CHINA. Yunnan, Lijiang, Yunshanping, on fallen trunk of Picea likiangensis, 18 June 1999, Dai 3089 (isotype, BJFC), on fallen trunk of *Abies*, 16 September 2018, Cui 17062 (BJFC), and on fallen trunk of *Picea*, 16 September 2018, Cui 17069 (BJFC).

***Dendroporia*** B.K. Cui & Xing Ji, gen. nov.

MycoBank: MB 847349

Differs from other genera by annual and resupinate basidiocarps with gray to pale brown pore surface, a dimitic hyphal system with weakly dextrinoid skeletal hyphae, tissues darkening in KOH, presence of dendrohyphidia and large rhomboid crystals, and hyaline to pale yellowish, thick-walled, ellipsoid, truncate, non-dextrinoid, and cyanophilous basidiospores.

Type species: *Dendroporia cinereofusca* (B.K. Cui & C.L. Zhao) B.K. Cui & Xing Ji

Etymology: *Dendroporia* (Lat.) refers to the presence of dendrohyphidia.

Basidiocarps are annual, resupinate, adnate, and corky. Pore surface is gray to pale brown. Subiculum is thin and clay buff to pale brown. Tubes are concolorous with pore surface and corky. Hyphal system is dimitic; generative hyphae with clamp connections; skeletal hyphae weakly dextrinoid and CB+; tissues are brown to black in KOH. Dendrohyphidia present at dissepimental edges; cystidia are absent; cystidioles are present. Large rhomboid crystals are present. Basidiospores ellipsoid, truncate, hyaline to pale yellowish, thick-walled, smooth, IKI−, and CB+.

Notes: In our present phylogeny, two specimens of *Perenniporia cinereofusca* B.K. Cui & C.L. Zhao formed a strongly supported clade distinct from the *Perenniporia* s. s. clade (Figure 1 and Figure 2). Morphologically, *P. cinereofusca* differs from species of *Perenniporia* s. s. by its resupinate basidiocarps with a gray to pale brown pore surface, tissues darkening in KOH, hyaline to pale yellowish, and non-dextrinoid basidiospores. Thus, the new genus *Dendroporia* is set up and the new combination *Dendroporia cinereofusca* is proposed.

In the current phylogenetic studies, *Dendroporia* is related to *Tropicoporia* and *Sparsitubus,* but with only weak support, and *Tropicoporia* differs from *Dendroporia* by its buff-yellow to grayish orange pore surface, non-dextrinoid skeletal hyphae, and dextrinoid basidiospores. *Sparsitubus* differs from *Dendroporia* in having effused reflexed to pileate basidiocarps and non-truncate, ornamented basidiospores [54].

***Dendroporia cinereofusca*** (B.K. Cui & C.L. Zhao) B.K. Cui & Xing Ji, comb. nov.

MycoBank: MB 847367

Basionym: *Perenniporia cinereofusca* B.K. Cui & C.L. Zhao, Mycoscience 55: 419 (2014).

For a detailed description of *Perenniporia cinereofusca*, see Zhao et al. [20].

Notes: *Dendroporia cinereofusca* was first described in *Perenniporia* from tropical China [20] and is characterized by its resupinate basidiocarps with gray to pale brown pore surfaces, a dimitic hyphal system with weakly dextrinoid skeletal hyphae, tissues becoming brown to black in KOH, the presence of dendrohyphidia, and hyaline to pale yellowish, truncate, and non-dextrinoid basidiospores.

Specimens examined: CHINA. Hainan, Ledong County, Jianfengling Nature Reserve, on fallen angiosperm trunk, 18 November 2007, Dai 9289 (holotype, BJFC); Lingshui County, Diaoluoshan Forest Park, on fallen angiosperm trunk, 20 November 2007, Cui 5280 (paratype, BJFC).

***Luteoperenniporia*** B.K. Cui & Xing Ji, gen. nov.

MycoBank: MB 847350

Differs from other genera by its resupinate basidiocarps with buff-yellow to cinnamon-buff pore surface, a dimitic hyphal system with weak to strong dextrinoid skeletal hyphae, the presence of cystidioles, and thick-walled, ellipsoid, and non-truncate, dextrinoid, and cyanophilous basidiospores.

Type species: *Luteoperenniporia bannaensis* (B.K. Cui & C.L. Zhao) B.K. Cui & Xing Ji

Etymology: *Luteoperenniporia* (Lat.) refers to resembling *Perenniporia* but with a buff-yellow pore surface when dry.

Basidiocarps are annual to perennial and resupinate. Pore surface iscream, buff to pale cinnamon buff when fresh, and becoming buff, buff-yellow to cinnamon-buff upon drying; pores are round to angular; dissepiments thin, entire to lacerate. Subiculum is thin and buff to cinnamon-buff. Tubes are concolorous with pore surface and corky. Hyphal system is dimitic, generative hyphae with clamp connections; skeletal hyphae weakly to strongly dextrinoid, CB+; tissues are unchanged in KOH. Cystidia is absent; cystidioles are present. Basidiospores are ellipsoid, non-truncate, hyaline, thick-walled, smooth, dextrinoid, and CB+.

Notes: In the combined ITS + nLSU and five-gene phylogenetic analyses, the species of *Luteoperenniporia* formed a single clade with high support (Figure 1 and Figure 2) distant from the *Perenniporia* s. s. clade. Morphologically, *Luteoperenniporia* differs from *Perenniporia* s. s. by its buff, buff-yellow to cinnamon-buff pore surfaces, a dimitic hyphal system with weakly to strongly dextrinoid skeletal hyphae and non-truncate basidiospores. Therefore, three new combinations are proposed in *Luteoperenniporia,* and the new species is described below.

***Luteoperenniporia bannaensis*** (B.K. Cui & C.L. Zhao) B.K. Cui & Xing Ji, comb. nov.

MycoBank: MB 847368

Basionym: *Perenniporia bannaensis* B.K. Cui & C.L. Zhao, Fungal Diversity 58: 52 (2013).

For a detailed description of *Perenniporia bannaensis*, see Zhao et al. [19].

Notes: *Luteoperenniporia bannaensis* was recently described in *Perenniporia* from China by Zhao et al. [19]; it is closely related to *L. yinggelingensis* in morphology and phylogeny; they share annual and resupinate basidiocarps, cream to buff pore surface and a dimitic hyphal system with dextrinoid and cyanophilous skeletal hyphae, and both species are distributed in the tropics. However, *L. yinggelingensis* is distinguished from *L. bannaensis* by its larger pores and basidiospores (pores 5–6 per mm, basidiospores 6.2–7.5 × 4.5–5.5 μm) [3].

Specimens examined: CHINA. Yunnan, Xishuangbanna, Mengla County, Wangtianshu Nature Reserve, on fallen angiosperm trunk, 2 November 2009, Cui 8560 (holotype, BJFC), Cui 8562 (paratype, BJFC).

***Luteoperenniporia mopanshanensis*** (C.L. Zhao) B.K. Cui & Xing Ji, comb. nov.

MycoBank: MB 847369

Basionym: *Perenniporia mopanshanensis* C.L. Zhao, Mycotaxon 134(1): 132 (2019).

For a detailed description of *Perenniporia mopanshanensis*, see Zhao and Ma [28].

Notes: *Luteoperenniporia mopanshanensis* was recently described in *Perenniporia* by Zhao and Ma [28]. *L. mopanshanensis* and *L. bannaensis* are both reported from Yunnan Province in southern China. They share resupinate basidiocarps, a dimitic hyphal system with strongly dextrinoid skeletal hyphae, non-truncate, and strongly dextrinoid and similar sized basidiospores. However, *L. bannaensis* differs by having an annual growth habit and smaller pores (6–8 per mm) [19].

***Luteoperenniporia yinggelingensis*** (B.K. Cui & Y.C. Dai) B.K. Cui & Xing Ji, comb. nov.

MycoBank: MB 847370

Basionym: *Perenniporia yinggelingensis* B.K. Cui & Y.C. Dai, Fungal Diversity 97: 300 (2019).

For a detailed description of *Perenniporia yinggelingensis*, see Cui et al. [3].

Notes: *Luteoperenniporia yinggelingensis* was newly described from a tropical area of China [3]. It is characterized by annual and resupinate basidiocarps with slightly lacerate pores, a distinct sterile margin, and a dimitic hyphal system with weakly dextrinoid skeletal hyphae. Macroscopically, *L. yinggelingensis* is close to *L. mopanshanensis*, but the latter species has perennial basidiocarps, larger pores (3–5 per mm), indistinct sterile margins, and strongly dextrinoid skeletal hyphae [28].

Specimens examined: CHINA. Hainan, Baisha County, Yinggeling Nature Reserve, on fallen angiosperm trunk, 17 November 2015, Cui 13625 (holotype, BJFC); on fallen angiosperm branch, 17 June 2016, Cui 13856 (BJFC).

**Figure 3:**
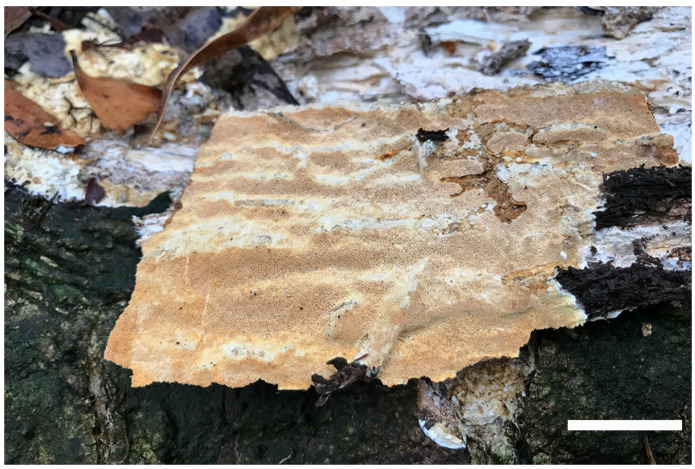
Basidiocarp of *Luteoperenniporia australiensis*. Scale bar = 2.0 cm.

**Figure 4:**
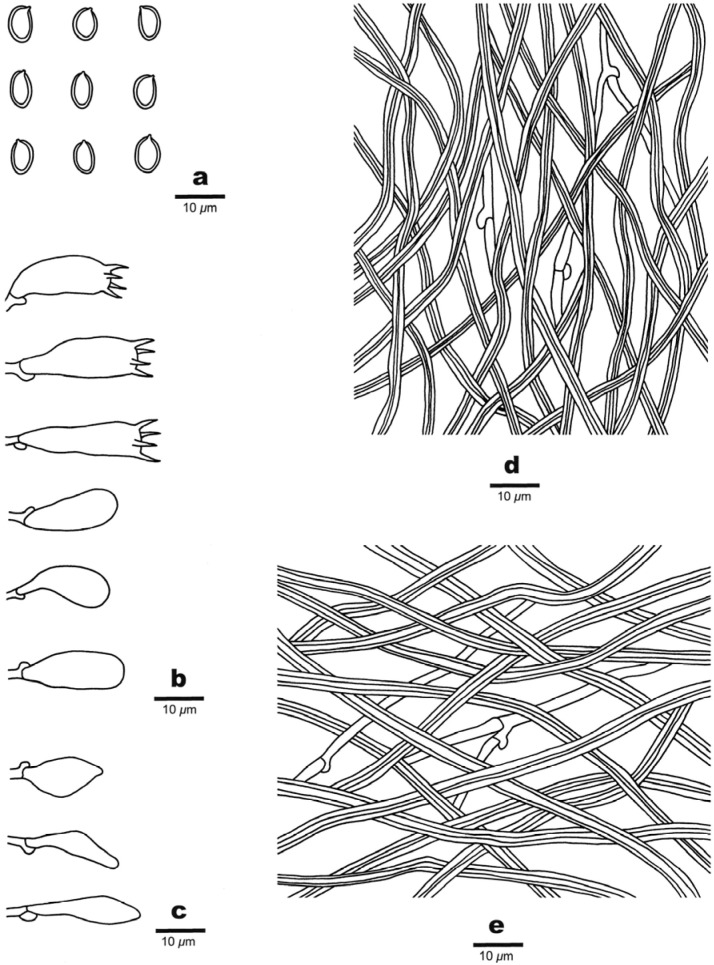
Microscopic structures of *Luteoperenniporia australiensis* (Holotype). (**a**). Basidiospores. (**b**). Basidia and basidioles. (**c**). Cystidioles. (**d**). Hyphae from trama. (**e**). Hyphae from subiculum.

***Luteoperenniporia australiensis*** B.K. Cui & Xing Ji, sp. nov.; Figure 3 and Figure 4

MycoBank: MB 847371

Differs from other species of *Luteoperenniporia* by its annual to perennial growth habit, resupinate basidiocarps with buff to cinnamon-buff pore surfaces, a dimitic hyphal system with dextrinoid skeletal hyphae, and ellipsoid, non-truncate, dextrinoid basidiospores (6.2–7.5 × 4–5.2 μm).

Holotype: AUSTRALIA. Tasmania, Keogh’s Creek Walk, on fallen trunk of *Eucalyptus*, 15 May 2018, Cui 16743 (BJFC).

Etymology: *australiensis* (Lat.) refers to the country where the new species was found.

Fruitbody: Basidiocarps are annual to perennial, resupinate, without odor or taste when fresh, corky to hard corky when dry, and up to 14 cm long, 8.5 cm wide, and 7 mm thick at center. Pore surface is buff-yellow, pinkish buff to pale cinnamon-buff when fresh, and buff, pale yellowish-brown to cinnamon-buff upon drying; pores are round to angular, 4–6 per mm, dissepiments thin, entire to lacerate. Sterile margin is distinct to indistinct, buff, and up to 2 mm wide. Subiculum is thin, buff, and up to 1 mm thick. Tubes are concolorous with pore surface, corky to hard corky when dry, and up to 6 mm long.

Hyphal structure: Hyphal system is dimitic; generative hyphae bearing clamp connections; skeletal hyphae dextrinoid, and CB+; tissues are unchanged in KOH.

Subiculum: Generative hyphae is infrequent, hyaline, thin-walled, occasionally branched, and 1.5–2.5 μm in diameter; skeletal hyphae is dominant, hyaline, thick-walled with a wide to narrow lumen, rarely branched, interwoven, and 2–5 μm in diameter.

Tubes: Generative hyphae is infrequent, hyaline, thin-walled, occasionally branched, and 1–2.4 μm in diameter; skeletal hyphae is dominant, hyaline, thick-walled with a wide to narrow lumen, occasionally branched, interwoven, and 1–3 μm. Cystidia are absent; fusoid cystidioles are present, hyaline, thin-walled, and 15.5–22 × 5.8–8 μm. Basidia clavate, with four sterigmata and a basal clamp connection, 15–24.5 × 6.5–9.7 μm; basidioles are dominant and in shape similar to basidia, but slightly smaller.

Spores: Basidiospores are ellipsoid, non-truncate, hyaline, thick-walled, smooth, dextrinoid, CB+, (6–) 6.2–7.4 (–7.7) × (3.9–) 4–5.2 (–5.4) μm, L = 6.77 μm, W = 4.58 μm, Q = 1.44–1.54 (n = 90/3).

Notes: *Luteoperenniporia australiensis* is characterized by its annual to perennial and resupinate basidiocarps, buff to cinnamon-buff pore surface, entire to lacerate pores, a dimitic hyphal system with dextrinoid skeletal hyphae, presence of cystidioles, and ellipsoid, non-truncate, dextrinoid, and cyanophilous basidiospores. *Luteoperenniporia bannaensis* is similar to *L. australiensis* by sharing a buff-yellow to pinkish buff pore surface, a dimitic hyphal system with dextrinoid, and cyanophilous skeletal hyphae. However, *L. bannaensis* differs from *L. australiensis* by its smaller pores (6–8 per mm) and smaller basidiospores (5.2–6 × 4–4.6 μm) [19]. *Luteoperenniporia yinggelingensis* may be confused with *L. australiensis* by having resupinate basidiocarps, and similar pore size and basidiospores. However, *L. yinggelingensis* is distinguished from *L. australiensis* mainly by its annual growth habit and cream to buff pore surface [3]. *Perenniporia subaurantiaca* (Rodway & Cleland) P.K. Buchanan & Ryvarden is also described from Tasmania, it is similar to *Luteoperenniporia australiensis* in its resupinate basidiocarps, dimitic hyphal system with dextrinoid and cyanophilous skeletal hyphae, and presence of cystidioles, but *P. subaurantiaca* has greyish cream to greyish orange pore surfaces and larger basidiospores (7.2–9.5 × 4.2–5.5 μm) [55]

Additional specimens (paratypes) examined: AUSTRALIA. Victoria, Yarra Ranges National Park, at the base of living *Eucalyptus*, 9 May 2018, Cui 16524 (BJFC), on fallen trunk of *Eucalyptus*, 9 May 2018, Cui 16525 (BJFC), 10 May 2018, Cui 16535 (BJFC), on living tree of *Eucalyptus*, 10 May 2018, Cui 16533, Cui 16534 (BJFC); Tasmania, Keogh’s Creek Walk, on fallen trunk of *Eucalyptus*, 15 May 2018, Cui 16742 (BJFC).

**Key to species of *Luteoperenniporia***

1. Pores 6–8 per mm……………………………………………………………………………………………………………………………………………*L. bannaensis*
1. Pores 3–6 per mm…………………………………………………………………………………………………………………………………………………………2
2. Skeletal hyphae unbranched…………………………………………………………………………………………………………………………*L. mopanshanensis*
2. Skeletal hyphae branched ……………………………………………………………………………………………………………………………………………… 3
3. Basidiocarps annual, distributed in tropical areas…………………………………………………………………………………………………*L. yinggelingensis*
3. Basidiocarps annual to perennial, distributed in temperate to subtropical areas…………………………………………………………………*L. australiensis*

***Macroporia*** B.K. Cui & Xing Ji, gen. nov.

MycoBank: MB 847352

Differs from other genera by its annual and resupinate basidiocarps, a dimitic hyphal system with dextrinoid skeletal hyphae, the presence of thin-walled cystidioles, and hyaline, thick-walled, ellipsoid, truncate, dextrinoid, and cyanophilous basidiospores.

Type species: *Macroporia macropora* (B.K. Cui & C.L. Zhao) B.K. Cui & Xing Ji

Etymology: *Macroporia* (Lat.) refers to the species with relatively large pores in *Perenniporia*.

Basidiocarps are annual, resupinate, adnate. Pore surface is white, cream to buff when fresh, and becoming buff, pinkish buff to yellowish buff upon drying; pores are angular; dissepiments are thin, entire to lacerate. Subiculum is thin, cream. Tubes are concolorous with pore surface, corky to hard corky. Hyphal system is dimitic, generative hyphae with clamp connections; skeletal hyphae branched, dextrinoid, CB+; tissues are unchanged in KOH. Cystidia is absent; cystidioles are usually present. Basidiospores are ellipsoid, truncate, hyaline, thick-walled, smooth, dextrinoid, and CB+.

Notes: In our ITS + nLSU and combined five-gene phylogenetic analyses, *Perenniporia lacerata* B.K. Cui & C.L. Zhao, *P. macropora* B.K. Cui & C.L. Zhao, *P. subrhizomorpha* Xue W. Wang, L.W. Zhou & X.M. Tian and *P. tibetica* B.K. Cui & C.L. Zhao grouped together and formed a well-supported clade (Figure 1 and Figure 2), which was distant from the *Perenniporia* s. s. clade. Morphologically, species in the clade usually have larger pores than those of *Perenniporia* s. s.; therefore, the new genus *Macroporia* is proposed to accommodate the four species.

***Macroporia lacerata*** (B.K. Cui & C.L. Zhao) B.K. Cui & Xing Ji, comb. nov.

MycoBank: MB 847372

Basionym: *Perenniporia lacerata* B.K. Cui & C.L. Zhao, Mycoscience 54: 232 (2013).

For a detailed description of *Perenniporia lacerata*, see Zhao and Cui [18].

Notes: *Macroporia lacerata* was originally described in *Perenniporia* from China by Zhao and Cui [18]. It is characterized by papery and thin basidiocarps, lacerate pores, a dimitic hyphal system with weakly dextrinoid skeletal hyphae, and ellipsoid, truncate, dextrinoid, and cyanophilous basidiospores.

Specimens examined: CHINA. Henan, Xiuwu County, Yuntaishan Park, on fallen angiosperm trunk, 3 September 2009, Cui 7220 (holotype, BJFC); Neixiang County, Baotianman Nature Reserve, on rotten angiosperm wood, 22 September 2009, Dai 11268 (paratype, BJFC).

***Macroporia macropora*** (B.K. Cui & C.L. Zhao) B.K. Cui & Xing Ji, comb. nov.

MycoBank: MB 847373

Basionym: *Perenniporia macropora* B.K. Cui & C.L. Zhao, Mycologia 105: 947 (2013).

For a detailed description of *Perenniporia macropora*, see Zhao and Cui [17].

Notes: *Macroporia macropora* was first described in *Perenniporia* from southern China by Zhao and Cui [17]. It is distinguished by large pores (2–3 per mm), a dimitic hyphal system with dextrinoid and branched skeletal hyphae, the presence of dendrohyphidia, and ellipsoid and truncate basidiospores. *M. macropora* is very close to *M. lacerata* in the current phylogeny, but *M. lacerata* differs from *M. macropora* by its smaller (3–5 per mm) and lacerate pores, the absence of dendrohyphidia, and smaller basidiospores (6.1–7 × 5–5.7 μm) [18].

Specimens examined: CHINA. Guangxi, Ningming County, Nonggang Nature Reserve, on fallen angiosperm branch, 8 July 2007, Zhou 407 (holotype, IFP), 7 July 2007, Zhou 297 (paratype, IFP).

***Macroporia subrhizomorpha*** (Xue W. Wang, L.W. Zhou & X.M. Tian) B.K. Cui & Xing Ji, comb. nov.

MycoBank: MB 847374

Basionym: *Perenniporia subrhizomorpha* Xue W. Wang, L.W. Zhou & X.M. Tian, Phytotaxa 528(2): 129 (2021).

For a detailed description of *Perenniporia subrhizomorpha*, see Tian et al. [56].

Notes: *Macroporia subrhizomorpha* was recently described in *Perenniporia* as *P. subrhizomorpha* by Tian et al. [56]. *Perenniporia rhizomorpha* may be confused with *P. subrhizomorpha* by sharing cream rhizomorphs and similar pore sizes, but the former species differs by its non-truncate basidiospores [10]. *Perenniporia tibetica* also has rhizomorphs but differs from *P. subrhizomorpha* by larger pores (2–3 per mm) [15].

***Macroporia tibetica*** (B.K. Cui & C.L. Zhao) B.K. Cui & Xing Ji, comb. nov.

MycoBank: MB 847375

Basionym: *Perenniporia tibetica* B.K. Cui & C.L. Zhao, Mycoscience 53: 366 (2012).

For a detailed description of *Perenniporia tibetica*, see Cui and Zhao [15].

Notes: *Macroporia tibetica* is characterized by resupinate basidiocarps with white to cream rhizomorphs, a dimitic hyphal system with slightly dextrinoid skeletal hyphae, and ellipsoid, truncate or not, dextrinoid, and cyanophilous basidiospores. *M. macropora* and *M. tibetica* share resupinate basidiocarps and similar pores, but the former differs in having dendrohyphidia and lacking rhizomorphs [17].

Specimens examined: CHINA. Xizang, Linzhi County, Tongmai, on fallen angiosperm trunk, 16 September 2010, Cui 9457 (holotype, BJFC), Cui 9459 (paratype, BJFC).

**Key to species of *Macroporia***

1. Pores lacerate …………………………………………………………………………………………………………………………………………………  *M. lacerata*
1. Pores entire ………………………………………………………………………………………………………………………………………………………………  2
2. Basidiocarps without rhizomorphs……………………………………………………………………………………………………………………… *M. macropora*
2. Basidiocarps with rhizomorphs…………………………………………………………………………………………………………………………………………3
3. Pores 2–3 per mm………………………………………………………………………………………………………………………………………………*M. tibetica*
3. Pores 4–6 per mm …………………………………………………………………………………………………………………………………… *M. subrhizomorpha*

***Macrosporia*** B.K. Cui & Xing Ji, gen. nov.

MycoBank: MB 847353

Differs from other genera by its annual and resupinate basidiocarps, cinnamon-buff pore surface, a trimitic hyphal system with weakly dextrinoid and cyanophilous skeletal hyphae, and hyaline, thick-walled, ellipsoid, truncate, strongly dextrinoid, and cyanophilous basidiospores.

Type species: *Macrosporia nanlingensis* (B.K. Cui & C.L. Zhao) B.K. Cui & Xing Ji

Etymology: *Macrosporia* (Lat.) refers to the large basidiospores.

Basidiocarps are annual, resupinate, adnate, corky when fresh, and becoming hard corky upon drying. Pore surface is cream-buff to yellowish buff when fresh, becoming cinnamon-buff upon drying; pores are round; dissepiments are thick, entire. Subiculum is cream to buff. Tubes are concolorous with the pore surface and hard corky. Hyphal system is trimitic; generative hyphae with clamp connections; skeletal and binding hyphae are weakly dextrinoid and CB+. Cystidia is absent; cystidioles are present. Basidiospores areellipsoid, truncate, hyaline, thick-walled, smooth, strongly dextrinoid, and CB+.

Notes: In our study, *Perenniporia nanlingensis* B.K. Cui & C.L. Zhao formed a single clade that was distant from the *Perenniporia* s. s. clade. Morphologically, it differs from *Perenniporia* s. s. by its annual and resupinate basidiocarps with cinnamon-buff pore surfaces and larger basidiospores. Therefore, *Macrosporia* gen. nov. is proposed to accommodate *P. nanlingensis*.

***Macrosporia nanlingensis*** (B.K. Cui & C.L. Zhao) B.K. Cui & Xing Ji, comb. nov.

MycoBank: MB 847376

Basionym: *Perenniporia nanlingensis* B.K. Cui & C.L. Zhao, Mycol. Prog. 11: 556 (2012).

For a detailed description of *Perenniporia nanlingensis*, see Zhao and Cui [16].

Notes: *Macrosporia nanlingensis* was first described in *Perenniporia* from southern China [16]; it is characterized by annual and resupinate basidiocarps, a cinnamon-buff pore surface when dry, a trimitic hyphal system with slightly dextrinoid skeletal hyphae, and ellipsoid, truncate, strongly dextrinoid, and cyanophilous basidiospores that are usually longer than 9 μm.

Specimens examined: CHINA. Guangdong Province, Ruyang County, Nanling Nature Reserve, on dead angiosperm tree, 16 September 2009, Cui 7589 (holotype, BJFC), Cui 7620 (paratype, BJFC).

***Minoporus*** B.K. Cui & Xing Ji, gen. nov.

MycoBank: MB 847354

Differs from other genera by its annual and pileate basidiocarps, cream to pale buff pileal surface when fresh, a dimitic hyphal system with weakly amyloid and cyanophilous skeletal hyphae, and hyaline, thick-walled, ellipsoid, truncate, dextrinoid, and cyanophilous basidiospores.

Type species: *Minoporus minor* (Y.C. Dai & H.X. Xiong) B.K. Cui & Xing Ji

Etymology: *Minoporus* (Lat.) refers to the small pilei.

Basidiocarps are annual, pileate, solitary, and soft corky when fresh, becoming hard corky upon drying. Pilei are semicircular to spathulate. Pileal surface is cream to pale buff when fresh, becoming cinnamon-buff when dry. Pore surface is cream when fresh, becoming cinnamon-buff when dry; pores are round. Context is white to cream, corky. Tubes are concolorous with pore surface and hard corky. Hyphal system dimitic; generative hyphae with clamp connections; skeletal hyphae is weakly amyloid and CB+. Cystidia and cystidioles are absent. Basidiospores are ellipsoid, truncate, hyaline, thick-walled, smooth, dextrinoid, and CB+.

Notes: In the ITS + nLSU analysis, two specimens of *Perenniporia minor* Y.C. Dai & H.X. Xiong formed a highly supported single clade (Figure 1), which is distinct from the *Perenniporia* s. s. clade and closely related to the *Perenniporiella* clade. Further phylogeny (Figure 2) inferred from the combined five-gene dataset indicated that the clade of *P. minor* was distant from the *Perenniporia* s. s. clade and closely related to the *Neoporia* clade. However, *Neoporia* has resupinate basidiocarps, dextrinoid skeletal hyphae, and non-truncate basidiospores; *Perenniporiella* has dextrinoid skeletal hyphae and non-truncate basidiospores [30]. *Perenniporia minor* differs from species of *Perenniporia* s. s. by its annual and pileate basidiocarps with a cream to pale buff pileal surface, a dimitic hyphal system with weakly amyloid skeletal hyphae. Therefore, *Minoporus* gen. nov. is proposed to accommodate *P. minor*.

***Minoporus minor*** (Y.C. Dai & H.X. Xiong) B.K. Cui & Xing Ji, comb. nov.

MycoBank: MB 847377

Basionym: *Perenniporia minor* Y.C. Dai & H.X. Xiong, Mycotaxon 105: 60 (2008).

For a detailed description of *Perenniporia minor*, see Xiong et al. [11].

Notes: This species was described from northeastern China by Xiong et al. [11] and is characterized by annual and pileate basidiocarps, cream to pale buff when fresh and cinnamon buff when dry pileal surface, a dimitic hyphal system with weakly amyloid skeletal hyphae, and ellipsoid, truncate, dextrinoid, and cyanophilous basidiospores.

Specimens examined: CHINA. Jilin, Antu County, Changbaishan Nature Reserve, Huangsongpu, on fallen branch of *Acer*, 14 September 2007, Dai 9198 (holotype, IFP); Liaoning, Huanren County, Laotudingzi Nature Reserve, on fallen branch of *Quercus*, 31 July 2008, Cui 5738 (BJFC).

***Neoporia*** B.K. Cui & Xing Ji, gen. nov.

MycoBank: MB 847355

Differs from other genera by its annual and resupinate basidiocarps with a buff-yellow pore surface, a dimitic hyphal system with dextrinoid and cyanophilous skeletal hyphae, and ellipsoid, non-truncate, dextrinoid, and cyanophilous basidiospores.

Type species: *Neoporia rhizomorpha* (B.K. Cui, Y.C. Dai & Decock) B.K. Cui & Xing Ji

Etymology: *Neoporia* (Lat.) refers to the genus resembling *Perenniporia*.

Basidiocarps are annual, resupinate and corky when dry. Pore surface is cream to buff when fresh, buff-yellow to grayish orange upon drying; pores are round to angular. Subiculum is cream to buff. Tubes are concolorous with the pore surface and corky. Hyphal system is dimitic; generative hyphae with clamp connections; skeletal hyphae dextrinoid, CB+; tissues unchanged in KOH. Basidiospores are ellipsoid, non-truncate, hyaline, thick-walled, smooth, dextrinoid, and CB+.

Notes: In our phylogenetic analyses, three species previously included in *Perenniporia*, *P. bostonensis* C.L. Zhao, *P. koreana* Y. Jang & J.J. Kim, and *P. rhizomorpha* B.K. Cui, Y.C. Dai & Decock grouped together and formed a highly supported clade (100% BS, 1.00 BPP, Figure 1; 100% BS, 1.00 BPP, Figure 2), which was distant from the *Perenniporia* s. s. clade. Morphologically, this clade differs from *Perenniporia* s. s. by its dimitic hyphal system and non-truncate basidiospores. Therefore, *Neoporia* gen. nov. is proposed to accommodate *P. bostonensis*, *P. koreana* and *P. rhizomorpha*.

In the five-gene phylogenetic analysis, *Neoporia* is related to *Luteoperenniporia*, *Minoporus*, *Poriella*, *Vanderbyliella,* and *Yuchengia*. *Luteoperenniporia*, *Neoporia*, *Poriella*, *Vanderbyliella,* and *Yuchengia* all have ellipsoid and non-truncate basidiospores. However, *Luteoperenniporia* differs from *Neoporia* in having a perennial growth habit; *Poriella* differs from *Neoporia* by having a perennial growth habit, cinnamon to ochraceous pore surfaces, and unbranched skeletal hyphae; *Vanderbyliella* is different from *Neoporia* by its pileate basidiocarps; *Yuchengia* differs from *Neoporia* in having amyloid skeletal hyphae and non-dextrinoid basidiospores [35]. In addition, *Minoporus* differs from *Neoporia* by its pileate basidiocarps, amyloid skeletal hyphae, and truncate basidiospores.

***Neoporia bostonensis*** (C.L. Zhao) B.K. Cui & Xing Ji, comb. nov.

MycoBank: MB 847378

Basionym: *Perenniporia bostonensis* C.L. Zhao, Phytotaxa 351(1): 67 (2018).

For a detailed description of *Perenniporia bostonensis*, see Shen et al. [27].

Notes: *Neoporia bostonensis* was recently described in *Perenniporia* from North America by Shen et al. [27]. It is characterized by resupinate basidiocarps with cream to buff pore surfaces, a dimitic hyphal system with strongly dextrinoid and unbranched skeletal hyphae, and ovoid to broad ellipsoid, non-truncate, and dextrinoid basidiospores.

***Neoporia koreana*** (Y. Jang & J.J. Kim) B.K. Cui & Xing Ji, comb. nov.

MycoBank: MB 847379

Basionym: *Perenniporia koreana* Y. Jang & J.J. Kim, Mycotaxon 130(1): 174 (2015).

For a detailed description of *Perenniporia bannaensis*, see Jang et al. [22].

Notes: *Neoporia koreana* was originally described from Republic of Korea as *Perenniporia koreana* by Jang et al. [22]; it has annual and resupinate basidiocarps with grayish orange pore surfaces, dextrinoid skeletal hyphae, and ellipsoid and non-truncate basidiospores. The sequences of *Neoporia koreana* from type specimens fell into *Neoporia* in our phylogeny. Thus, *P. koreana* is transferred to *Neoporia*. *N. koreana* is similar to *N. bostonensis* in resupinate basidiocarps, similar sized pores, dextrinoid skeletal hyphae, and non-truncate basidiospores, but the former has larger basidiospores (6–7 × 3.9–5.2 μm) [22] and the latter has smaller basidiospores (3.5–4.5 × 3–4 μm) [27].

***Neoporia rhizomorpha*** (B.K. Cui, Y.C. Dai & Decock) B.K. Cui & Xing Ji, comb. nov.

MycoBank: MB 847380

Basionym: *Perenniporia rhizomorpha* B.K. Cui, Y.C. Dai & Decock, Mycotaxon 99: 176 (2007).

For a detailed description of *Perenniporia rhizomorpha*, see Cui et al. [10].

Notes: *Neoporia rhizomorpha* was first described in *Perenniporia* based on morphological characters from China [10]. It is unique in *Neoporia* due to its resupinate basidiocarps with rhizomorphs.

Specimens examined: CHINA. Anhui, Huangshan, Yellow Mountain, on fallen angiosperm trunk, 13 October 2004, Dai 6165 (holotype, BJFC); Fujian, Wuyishan County, Wuyishan Nature Reserve, on fallen angiosperm branch, 19 October 2005, Cui 7248 (paratype, BJFC).

**Key to species of *Neoporia***

1. Basidiocarps with rhizomorphs…………………………………………………………………………………………………………………………*N. rhizomorpha*
1. Basidiocarps without rhizomorphs…………………………………………………………………………………………………………………………………… 2
2. Basidiospores 3.5–4.5 × 3–4 μm ………………………………………………………………………………………………………………………… *N. bostonensis*
2. Basidiospores 6–7 × 3.9–5.2 μm………………………………………………………………………………………………………………………………*N. koreana*

***Niveoporia*** B.K. Cui & Xing Ji, gen. nov.

MycoBank: MB 847356

Differs from other genera by perennial basidiocarps with white pore surface when fresh, distinct rusty red to reddish brown sterile margin, a dimitic hyphal system with dextrinoid and cyanophilous skeletal hyphae, the presence of cystidioles, hyaline, and thick-walled, ellipsoid, and truncate basidiospores.

Type species: *Niveoporia russeimarginata* (B.K. Cui & C.L. Zhao) B.K. Cui & Xing Ji

Etymology: *Niveoporia* (Lat.) refers to the white pore surface.

Basidiocarps are perennial, resupinate to pileate, corky to woody hard when dry. Pilei dimidiate to fan shaped. Pileal surface is clay-buff to reddish brown when fresh, grayish brown to umber brown when dry, glabrous, and concentrically sulcate. Pore surface is white when fresh and white to cream upon drying; pores are round. The sterile margin is sometimes distinct, rusty red to reddish brown. Context is buff to fawn. Tubes are cream to pale cinnamon. Hyphal system is dimitic; generative hyphae with clamp connections; skeletal hyphae dextrinoid, CB+; tissues unchanged in KOH. Cystidia is absent; cystidioles are present. Basidiospores are ellipsoid, truncate, hyaline, thick-walled, smooth, dextrinoid or not, and CB+.

Notes: In the phylogenetic analysis of this study, *P. decurrata* F. Wu & X.H. Ji, *P. russeimarginata* B.K. Cui & C.L. Zhao and an undescribed species grouped together and formed a well-supported clade (74% BS, 1.00 BPP, Figure 1; 83% BS, 0.97 BPP, Figure 2) which was separated from the *Perenniporia* s. s. clade. Morphologically, species in the clade usually have pileate basidiocarps and white pore surfaces with reddish brown sterile margins, which are different from the species of *Perenniporia* s. s. Thus, *Niveoporia* gen. nov. is proposed to accommodate these species.

***Niveoporia decurrata*** (Corner) B.K. Cui & Xing Ji, comb. nov.

MycoBank: MB 847381

Basionym: *Perenniporia decurrata* Corner, Beih. Nova Hedwigia 96: 105 (1989)

= *Perenniporia chiangraiensis* F. Wu & X.H. Ji, Mycosphere 8(8): 1103 (2017).

For a detailed description of *Perenniporia decurrata*, see Corner [52].

Notes: *Perenniporia decurrata* was first described from Malaysia [52]. *Perenniporia chiangraiensis* was recently described from Northern Thailand based on morphological characters and molecular data by Ji et al. [25]. However, these authors overlooked *P. decurrata*, which is a priority name for this species. The species is characterized by pileate basidiocarps with concentrically sulcate pileal surfaces, white pore surfaces, the presence of dendrohyphidia and cystidioles, and ellipsoid, truncate, thick-walled, and non-dextrinoid basidiospores.

Specimens examined: CHINA. Yunnan, Xishuangbanna, Menglun, on fallen angiosperm trunk, 12 September 2006, Yuan 2334 (IFP). THAILAND. Chiang Rai, Doi Mae Salong, on angiosperm tree root, 22 July 2016, Dai 16637 (BJFC).

***Niveoporia russeimarginata*** (B.K. Cui & C.L. Zhao) B.K. Cui & Xing Ji, comb. nov.

MycoBank: MB 847382

Basionym: *Perenniporia russeimarginata* B.K. Cui & C.L. Zhao, Mycologia 105(4): 947 (2013).

For a detailed description of *Perenniporia russeimarginata*, see Zhao and Cui [17].

Notes: *Niveoporia russeimarginata* was first described in *Perenniporia* from southern China [17]. *Perenniporia alboferruginea* Decock, described from Cameroon in Africa, is similar to *N. russeimarginata* in having resupinate basidiocarps with ferruginous red upper margins and a dimitic hyphal system. However, *P. alboferruginea* differs by having an annual growth habit, larger pores (5–6 per mm), the absence of cystidioles, and non-dextrinoid basidiospores [57].

Specimens examined: CHINA. Yunnan, Chuxiong, Zixishan Nature Reserve, on fallen angiosperm trunk, 1 August 2005, Yuan 1225 (holotype, IFP), Yuan 1262 (paratype, IFP).

**Figure 5:**
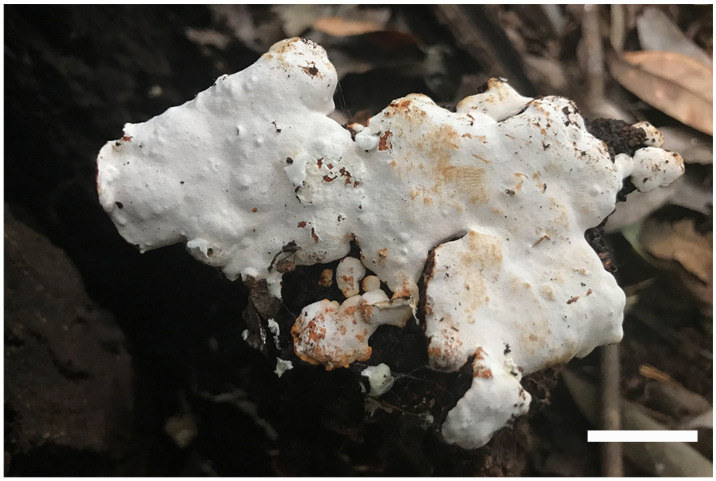
Basidiocarp of *Niveoporia subrusseimarginata* (Holotype). Scale bar = 2.0 cm.

**Figure 6:**
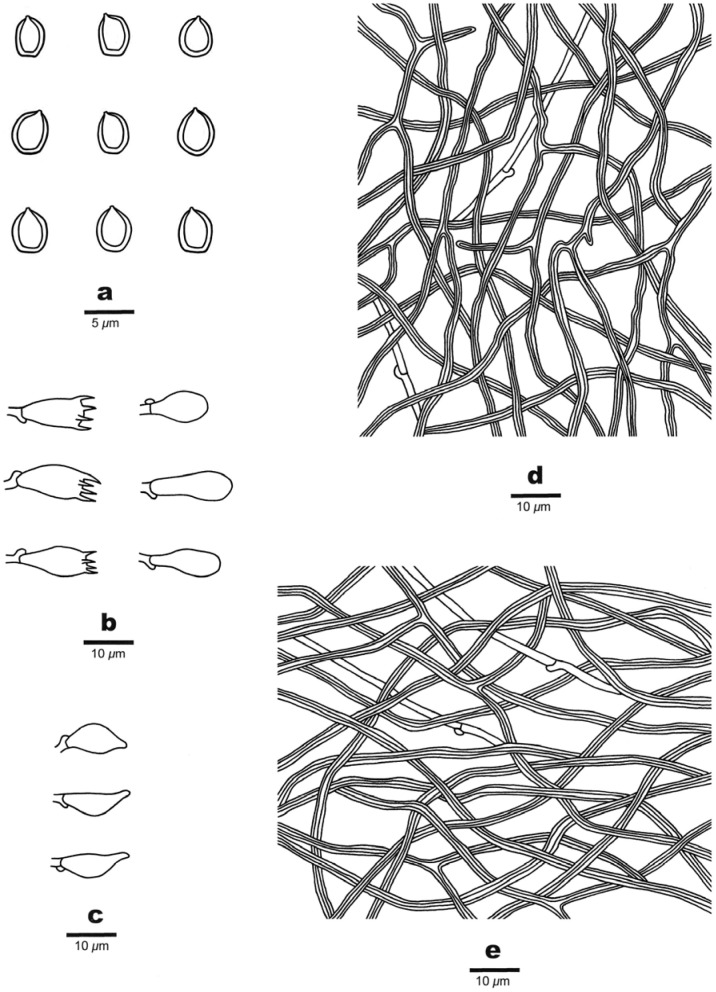
Microscopic structures of *Niveoporia subrusseimarginata* (Holotype). (**a**). Basidiospores. (**b**). Basidia and basidioles. (**c**). Cystidioles. (**d**). Hyphae from trama. (**e**). Hyphae from context.

***Niveoporia subrusseimarginata*** B.K. Cui & Xing Ji, sp. nov., Figure 5 and Figure 6

MycoBank: MB 847383

Differs from other *Niveoporia* species by its resupinate to pileate basidiocarps with rusty reddish brown sterile margins and pores measuring 5–6 per mm.

Holotype: CHINA. Yunnan, Binchuan County, Jizushan Park, on angiosperm stump, 14 September 2018, Cui 16991 (BJFC).

Etymology: *Subrusseimarginata* (Lat.) refers to the species resembling *Niveoporia russeimarginata*.

Fruitbody: Basidiocarps are perennial, resupinate, sometimes pileate, corky, without odor or taste when fresh, becoming woody hard upon drying. Pilei are irregular, projecting up to 2 cm, 5 cm wide, and 3 cm thick at the base; with resupinate up to 9 cm long, 5 cm wide, and 1.3 cm thick at center. Pileal surface is orange-brown to reddish brown when fresh, umber brown when dry, glabrous, and concentrically sulcate. The pore surface is white when fresh, cream upon drying; pores are round to angular, 5–6 per mm, dissepiments thick, entire. Sterile margin is distinct to indistinct, cinnamon to rusty reddish brown, and up to 2 mm wide. Subiculum is buff, thin, up to 0.5 mm thick. Tubes are buff, woody hard when dry, and up to 11.5 mm long.

Hyphal structure: Hyphal system is dimitic; generative hyphae bearing clamp connections; skeletal hyphae is weakly dextrinoid and CB+; tissues are unchanged in KOH.

Subiculum: Generative hyphae are infrequent, hyaline, thin-walled, unbranched, and 1–2.2 μm in diameter; skeletal hyphae are dominant, hyaline, thick-walled, occasionally branched, interwoven, and 1–2.5 μm in diameter.

Tubes: Generative hyphae are infrequent, hyaline, thin-walled, occasionally branched, and 1–2 μm in diameter; skeletal hyphae are dominant, hyaline, thick-walled, moderately branched, and interwoven, 1–2.3 μm. Cystidia are absent; fusoid cystidioles are present, hyaline, thin-walled, and 12.5–14 × 4.8–6 μm. Basidia are clavate, with four sterigmata and a basal clamp connection, 12.5–17.5 × 6.3–9 μm; basidioles are dominant and in shape similar to basidia, but slightly smaller.

Spores: Basidiospores are broadly ellipsoid, truncate or not, hyaline, thick-walled, smooth, weakly dextrinoid, CB+, (4–)4.2–4.8 (–5) × (3–)3.2–3.8(–4) μm, L = 4.48 μm, W = 3.51 μm, Q = 1.27–1.3 (n = 90/3).

Notes: *Niveoporia subrusseimarginata* is characterized by resupinate to pileate basidiocarps with white to cream pore surface, cinnamon to rust sterile margin, dimitic hyphal system with weakly dextrinoid skeletal hyphae, the presence of cystidioles, and broadly ellipsoid, truncate or not basidiospores.

Additional specimens (paratypes) examined: CHINA. Yunnan, Binchuan County, Jizushan Park, on the stump of *Quercus*, 14 September 2018, Cui 16973 (BJFC), on fallen angiosperm branch, 14 September 2018, Cui 16980 (BJFC), on angiosperm stump, 14 September 2018, Cui 16988, 16990 (BJFC).

**Key to species of *Niveoporia***

1. Sterile margin indistinct …………………………………………………………………………………………………………………………………… *N. decurrata*
1. Sterile margin distinct, reddish brown…………………………………………………………………………………………………………………………………2
2. Pores 5–6 per mm………………………………………………………………………………………………………………………………… *N. subrusseimarginata*
2. Pores 6–8 per mm …………………………………………………………………………………………………………………………………… *N. russeimarginata*

***Perenniporia*** Murrill, Mycologia 34(5): 595 (1942).

MycoBank: MB 18204

Type species: *Perenniporia medulla-panis* (Jacq.) Donk, Persoonia 5(1): 76 (1967).

Basidiocarps are annual to perennial and resupinate. Pore surface is white to cream when fresh, cream to buff when dry; pores are round and small. Subiculum is thin, cream, and corky. Tubes are concolorous with pore surface and corky. Hyphal system is dimitic to trimitic; generative hyphae with clamp connections; skeletal hyphae are non-dextrinoid to dextrinoid or amyloid, cyanophilous; tissues are unchanged in KOH. Cystidia are absent, cystidioles are present. Basidiospores are ellipsoid, truncate, hyaline, thick-walled, smooth, dextrinoid, and CB+.

Notes: In our phylogenetic analyses, the species of *Perenniporia* s. l. clustered into several clades, *Perenniporia medulla-panis* is grouped with *P. substraminea* B.K. Cui & C.L. Zhao and *P. hainaniana* B.K. Cui & C.L. Zhao. These three species share similar morphological characteristics and form the core clade of *Perenniporia*. The above concept of *Perenniporia* s. s. is determined from *P. medulla-panis*, *P. hainaniana,* and *P. substraminea*.

Specimens examined: *Perenniporia hainaniana*. China. Hainan, Changjiang County, Bawangling Nature Reserve, on angiosperm stump, 8 May 2009, Cui 6364 (holotype, BJFC). *Perenniporia medulla-panis*. China. Guangxi, Jinxiu County, Dayaoshan Nature Reserve, on living angiosperm tree, 15 July 2017, Cui 14515 (BJFC). *Perenniporia substraminea*. China. Zhejiang, Taishun County, Wuyanling Nature Reserve, on angiosperm stump, 22 August 2011, Cui 10177 (holotype, BJFC).

**Key to species of *Perenniporia* s. s.**

1. Pores > 8 per mm…………………………………………………………………………………………………………………………………………*P. substraminea*
1. Pores < 7 per mm…………………………………………………………………………………………………………………………………………………………2
2. Dendrohyphidia present at dissepimental edges………………………………………………………………………………………………………*P. hainaniana*
2. Dendrohyphidia absent at dissepimental edges…………………………………………………………………………………………………… *P. medulla-panis*

***Poriella*** C.L. Zhao, Agronomy 11(7, no. 1308): 5 (2021).

MycoBank: MB 840061

Type species: *Poriella subacida* (Peck) C.L. Zhao, Agronomy 11(7, no. 1308): 6 (2021).

Basidiocarps are annual to perennial, resupinate to effused-reflexed, and corky when dry. Pore surface is dingy yellowish, cinnamon to ochraceous; pores are round to angular. Context is thin, cream, buff to pale ochraceous. Tubes are concolorous with pore surface, corky. Hyphal system is dimitic to trimitic; generative hyphae with clamp connections; skeletal hyphae is unbranched, strongly dextrinoid, and cyanophilous. Basidiospores are ellipsoid to subglobose, non-truncate, hyaline, thick-walled, smooth, non-dextrinoid to dextrinoid, and CB+.

Notes: *Poriella* was newly set up by Chen et al. [37] according to analyses of ITS, nLSU, mtSSU, and TEF1 datasets. The type species *Poriella subacida*, originally described as *Polyporus subacidus* Peck [58], was usually treated in *Perenniporia* in current studies [2,3,59]. In our phylogeny, three species of *Perenniporia*, *P. africana* Ipulet & Ryvarden, *P. ellipsospora* Ryvarden & Gilb., and *P. valliculorum* Spirin et Zmitr. grouped together with *Poriella subacida* and formed a highly supported clade (100% BS, 1.00 BPP, Figure 1; 98% BS, 1.00 BPP, Figure 2); morphologically, these three species have resupinate basidiocarps, a dimitic hyphal system with unbranched and strongly dextrinoid skeletal hyphae, and hyaline, thick-walled, and non-truncate basidiospores, which are quite consistent with the concept of *Poriella*. Therefore, these three species are transferred to *Poriella* based on molecular data and morphological characters.

***Poriella africana*** (Ipulet & Ryvarden) B.K. Cui & Xing Ji, comb. nov.

MycoBank: MB 847384

Basionym: *Perenniporia africana* Ipulet & Ryvarden, Syn. Fung. 20: 94 (2005).

For a detailed description of *Perenniporia africana*, see Ipulet and Ryvarden [60] and Cui et al. [3].

Notes: *Poriella africana* was originally described from Uganda as *Perenniporia africana* [60]. It has perennial and resupinate basidiocarps, a dimitic hyphal system with unbranched and strongly dextrinoid skeletal hyphae, and subglobose to broadly ellipsoid and non-truncate basidiospores.

Specimens examined: CHINA. Anhui, She County, Qingliangfeng Nature Reserve, on fallen angiosperm trunk, 14 December 2009, Cui 8674, 8676 (BJFC).

***Poriella ellipsospora*** (Ryvarden & Gilb.) B.K. Cui & Xing Ji, comb. nov.

MycoBank: MB 847385

Basionym: *Perenniporia ellipsospora* Ryvarden & Gilb., Mycotaxon 19: 140 (1984).

For a detailed description of *Perenniporia ellipsospora*, see Cui et al. [3].

Notes: *Poriella ellipsospora* and *P. subacida* both have resupinate basidiocarps, strongly dextrinoid skeletal hyphae, and ellipsoid and non-truncate basidiospores, but the former has larger pores (3–4 per mm) and dextrinoid basidiospores, and the latter has smaller pores (4–6 per mm) and non-dextrinoid basidiospores [37].

Specimens examined: CHINA. Yunnan, Lanping County, Changyanshan Nature Reserve, on fallen angiosperm trunk, 18 September 2011, Cui 10276, 10284 (BJFC).

***Poriella valliculorum*** (Spirin & Zmitr.) B.K. Cui & Xing Ji, comb. nov.

Mycobank: MB 847386

Basionym: *Perenniporia valliculorum* Spirin & Zmitr., Folia Cryptogamica Petropolitana (Sankt-Peterburg) 6: 51 (2005).

For a detailed description of *Perenniporia valliculorum*, see Spirin et al. [9].

Notes: *Poriella valliculorum* was originally described from Russia as *Perenniporia valliculorum* by Spirin et al. [9]. It has a pale citric yellow to pale tan pore surface, strongly dextrinoid skeletal hyphae, and non-truncate basidiospores, and these characters fit *Poriella* well. In addition, the sequence of *P. valliculorum* from the type specimen fell into *Poriella* in our phylogeny (Figure 1 and Figure 2). Therefore, *Perenniporia valliculorum* is transferred to *Poriella*.

**Key to species of *Poriella***

1. Basidiospores non-dextrinoid ………………………………………………………………………………………………………………………………  *P. subacida*
1. Basidiospores dextrinoid…………………………………………………………………………………………………………………………………………………2
2. Pores < 5 per mm …………………………………………………………………………………………………………………………………………  *P. ellipsospora*
2. Pores > 5 per mm………………………………………………………………………………………………………………………………………………………… 3
3. Basidiocarps perennial…………………………………………………………………………………………………………………………………………*P. africana*
3. Basidiocarps annual to perennial ……………………………………………………………………………………………………………………… *P. valliculorum*

***Rhizoperenniporia*** B.K. Cui & Xing Ji, gen. nov.

MycoBank: MB 847357

Differs from other genera by its resupinate basidiocarps with rhizomorphs, a dimitic hyphal system with weakly dextrinoid skeletal hyphae, and hyaline, thick-walled, ellipsoid, truncate, dextrinoid, and cyanophilous basidiospores.

Type species: *Rhizoperenniporia japonica* (Yasuda) B.K. Cui & Xing Ji

Etymology: *Rhizoperenniporia* (Lat.) refers to resembling *Perenniporia* but with rhizomorphs.

Basidiocarps are annual to perennial, resupinate, and corky when dry. Rhizomorphs are present. Pore surface is grayish white to pale buff when dry; pores are round; dissepiments thick, entire. Subiculum is thin, cream. Tubes are concolorous with pore surface and corky. Hyphal system is dimitic; generative hyphae with clamp connections; skeletal hyphae are dextrinoid and CB+; tissues are unchanged in KOH. Cystidia are absent; cystidioles are present. Basidiospores are ellipsoid, truncate, hyaline, thick-walled, smooth, dextrinoid, and CB+.

Notes: In our phylogenetic analyses (Figure 1 and Figure 2), two specimens of *Perenniporia japonica* (Yasuda) T. Hatt. & Ryvarden formed a single clade distant from the *Perenniporia* s. s. clade. In addition, *P. japonica* has basidiocarps with rhizomorphs, which are different from other species of *Perenniporia* s. s. Therefore, the new genus *Rhizoperenniporia* is proposed to include *P. japonica*.

***Rhizoperenniporia japonica*** (Yasuda) B.K. Cui & Xing Ji, comb. nov.

MycoBank: MB 847387

Basionym: *Trametes japonica* Yasuda, Bot. Mag., Tokyo 32: 356 (1918).

= *Perenniporia japonica* (Yasuda) T. Hatt. & Ryvarden, Mycotaxon 50: 36 (1994).

For a detailed description of *Perenniporia japonica*, see Núñez and Ryvarden [2] and Cui et al. [3].

Notes: In *Perenniporia* s. l., *P. aurantiaca*, *P. bambusicola*, *P. rhizomorpha*, *P. subrhizomorpha,* and *P. tibetica* also have resupinate basidiocarps with rhizomorphs. However, *P. aurantiaca* differs from *P. japonica* by an orange pore surface, arboriform vegetative hyphae, and tissues becoming violet in KOH [4]. *Perenniporia bambusicola* is distinguished by an orange pore surface turning dark violet to black in KOH, arboriform vegetative hyphae, and growing only on bamboo [51]. *Perenniporia rhizomorpha* differs by its non-truncate basidiospores [10]. *Perenniporia subrhizomorpha* differs by the absence of cystidioles and larger basidiospores (5.7–6.5 × 4.3–5.5 μm) [56]. *Perenniporia tibetica* differs by having larger pores and basidiospores (pores 2–3 per mm, basidiospores 6.7–8.7 × 5.3–6.8 μm) [15].

Specimens examined: CHINA. Shanxi, Yangcheng County, Manghe Nature Reserve, on rotten wood of *Vitex*, 25 August 2016, Dai 17035 (BJFC); Huguan County, Baquan Gorge, on fallen trunk of *Lonicera*, 27 August 2016, Dai 17068, 17080 (BJFC).

***Tropicoporia*** B.K. Cui & Xing Ji, gen. nov.

MycoBank: MB 847358

Differs from other genera by its dimitic to trimitic hyphal system with usually non-dextrinoid and inamyloid skeletal hyphae, broadly ellipsoid to subglobose, truncate, dextrinoid, and cyanophilous basidiospores.

Type species: *Tropicoporia aridula* (B.K. Cui & C.L. Zhao) B.K. Cui & Xing Ji

Etymology: *Tropicoporia* (Lat.) refers to the distribution of the genus in tropical areas.

Basidiocarps are annual to perennial, mostly resupinate, and pseudopileate to rarely pileate. Pore surface is cream, buff-yellow to grayish orange; pores are round to angular. Hyphal system is dimitic to trimitic; generative hyphae with clamp connections; skeletal hyphae are non-dextrinoid to slightly dextrinoid and CB+. Basidiospores are broadly ellipsoid to subglobose, truncate, hyaline, thick-walled, smooth, dextrinoid, and CB+.

Notes: The phylogenies inferred from the two combined datasets ITS + nLSU and ITS + nLSU + mtSSU + TEF1 + TBB1 showed that *Perenniporia aridula* B.K. Cui & C.L. Zhao, *P. vanhulleae* Decock & Ryvarden, *P. centrali-africana* Decock & Mossebo, and *P. brasiliensis* C.R.S. de Lira et al. grouped together and formed a clade distinct from the *Perenniporia* s. s. clade, although the branch support was low (Figure 1 and Figure 2). Morphologically, the four species usually have non-dextrinoid skeletal hyphae and broadly ellipsoid to subglobose basidiospores, which are different from the species of *Perenniporia* s. s.. Thus, *Tropicoporia* gen. nov. is proposed to accommodate the four species.

In our study, *Tropicoporia* was closely related to *Sparsitubus* and then grouped with *Dendroporia* with low support (Figure 1 and Figure 2). Morphologically, *Sparsitubus* differs from *Tropicoporia* by its effused-reflexed to pileate basidiocarps, and asperulate and non-truncate basidiospores [54]. *Dendroporia* is distinguished from *Tropicoporia* by its gray to pale brown pore surface, dextrinoid skeletal hyphae, and non-dextrinoid basidiospores.

***Tropicoporia aridula*** (B.K. Cui & C.L. Zhao) B.K. Cui & Xing Ji, comb. nov.

MycoBank: MB 847388

Basionym: *Perenniporia aridula* B.K. Cui & C.L. Zhao, Fungal Diversity 58: 48 (2013)

For a detailed description of *Perenniporia aridula*, see Zhao et al. [19].

Notes: *Tropicoporia aridula* was first described in *Perenniporia* by Zhao et al. [19]. It is thus far known only from Southwest China. *Tropicoporia vanhulleae* is closely related to *T. aridula* in morphology and phylogeny, but the former has smaller basidiospores (5.5–6.0 × 4.5–5.5 µm) [21].

Specimens examined: CHINA. Yunnan, Yuanjiang County, on fallen angiosperm trunk, 9 June 2011, Dai 12396 (holotype, BJFC), on fallen bamboo, 9 June 2011, Dai 12398 (paratype, BJFC).

***Tropicoporia brasiliensis*** (Lira, A.M.S. Soares, Ryvarden & Gibertoni) B.K. Cui & Xing Ji, comb. nov.

MycoBank: MB 847389

Basionym: *Perenniporia brasiliensis* Lira, A.M.S. Soares, Ryvarden & Gibertoni, Persoonia 38: 355 (2017).

For a detailed description of *Perenniporia brasiliensis*, see Lira et al in Crous et al. [23].

Notes: *Tropicoporia brasiliensis* was originally described from Brazil as *Perenniporia* brasiliensis by Lira et al (in Crous et al. [23]); it has subglobose to globose and small basidiospores (3–4 × 2–4 μm) [23].

***Tropicoporia centrali-africana*** (Decock & Mossebo) B.K. Cui & Xing Ji, comb. nov.

MycoBank: MB 847390

Basionym: *Perenniporia centrali-africana* Decock & Mossebo, Systematics and Geography of Plants 71(2): 608 (2002).

For a detailed description of *Perenniporia centrali-africana*, see Decock and Mossebo [61].

Notes: *Tropicoporia centrali-africana* was originally described from Cameroon as *Perenniporia centrali-africana* [61]. Lira et al (in Crous et al. [23]) also reported this species from Brazil. It differs from the other three species by its pileate basidiocarps.

***Tropicoporia vanhulleae*** (Decock & Ryvarden) B.K. Cui & Xing Ji, comb. nov.

MycoBank: MB 847391

Basionym: *Perenniporia vanhulleae* Decock & Ryvarden, Index Fungorum 234: 1 (2015)

For a detailed description of *Perenniporia vanhulleae*, see Decock and Ryvarden [21].

Notes: *Tropicoporia vanhulleae* was originally described from Africa as *Perenniporia vanhulleae* by Decock and Ryvarden [21]. The sequence of *T. vanhulleae* from the type specimens fell into *Tropicoporia* in our phylogeny. Therefore, *Perenniporia vanhulleae* is transferred to *Tropicoporia*.

**Key to species of *Tropicoporia***

1. Basidiocarps resupinate to pileate………………………………………………………………………………………………………………… *T. centrali-africana*
1. Basidiocarps resupinate…………………………………………………………………………………………………………………………………………………2
2. Basidiospores < 5 µm………………………………………………………………………………………………………………………………………*T. brasiliensis*
2. Basidiospores > 5 µm……………………………………………………………………………………………………………………………………………………3
3. Basidiospores 6–7 μm………………………………………………………………………………………………………………………………………… *T. aridula*
3. Basidiospores 5.5–6.0 µm………………………………………………………………………………………………………………………………… *T. vanhulleae*

***Truncatoporia*** B.K. Cui & Xing Ji, gen. nov.

MycoBank: MB 847359

Differs from other genera by its resupinate to pileate basidiocarps, a dimitic to trimitic hyphal system with dextrinoid and cyanophilous skeletal hyphae, and thick-walled, ellipsoid, truncate, and cyanophilous basidiospores.

Type species: *Truncatoporia truncatospora* (Lloyd) B.K. Cui & Xing Ji

Etymology: *Truncatoporia* (Lat.) refers to the truncate basidiospores of the genus.

Basidiocarps are annual to perennial, resupinate to pileate, and corky. Pileal surface is brown to ochraceous. Pore surface is buff to pale yellowish buff upon drying; pores are round to angular; dissepiments thin, entire. Context cream buff to pale brown. Tubes are concolorous with pore surface. Hyphal system is dimitic to trimitic; generative hyphae with clamp connections; skeletal hyphae are dextrinoid and CB+; tissues are unchanged in KOH. Basidiospores are ellipsoid, truncate, hyaline, thick-walled, smooth, dextrinoid or not, and CB+.

Notes: In our phylogenetic analyses, two species previously included in *Perenniporia*, *P. pyricola* Y.C. Dai & B.K. Cui and *P. truncatospora* (Lloyd) Ryvarden, grouped together and formed a well-supported clade (78% BS, 1.00 BPP, Figure 1; 98% BS, 1.00 BPP, Figure 2) distinct from the *Perenniporia* s. s. clade. Therefore, *Truncatoporia* gen. nov. is proposed to accommodate *P. pyricola* and *P. truncatospora*.

***Truncatoporia pyricola*** (Y.C. Dai & B.K. Cui) B.K. Cui & Xing Ji, comb. nov.

MycoBank: MB 847392

Basionym: *Perenniporia pyricola* Y.C. Dai & B.K. Cui, Mycosystema 29(6): 815 (2010).

For a detailed description of *Perenniporia pyricola*, see Dai [62].

Notes: *Truncatoporia pyricola* was first described in *Perenniporia* from Northeast China [62]. It has a distribution in northern China mainly on *Pyrus* and *Prunus*. The species has perennial and resupinate basidiocarps with cream to pale cinnamon, a dimitic hyphal system with dextrinoid and cyanophilous skeletal hyphae, thick-walled and truncate, dextrinoid, and cyanophilous basidiospores.

Specimens examined: CHINA. Liaoning, Anshan, Qianshan Park, on living tree of *Pyrus*, 2 August 2008, Dai 10265 (holotype, BJFC); Tianjin, Ji County, Panshan Mountain, Living tree of *Crataegus*, 6 August 2015, Dai 15496 (BJFC), Dai 15498 (BJFC).

***Truncatoporia truncatospora*** (Lloyd) B.K. Cui & Xing Ji, comb. nov.

MycoBank: MB 847393

Basionym: *Trametes truncatospora* Lloyd, Mycol. Writ. 6: 853 (1919).

= *Perenniporia truncatospora* (Lloyd) Ryvarden, Acta Mycol. Sin. 5: 228 (1986).

For a detailed description of *Perenniporia truncatospora*, see Cui et al. [3].

Notes: *Truncatoporia truncatospora* and *T. pyricola* both have dextrinoid skeletal hyphae and truncate basidiospores, but the former has pileate basidiocarps, smaller pores (6–8 per mm), and non-dextrinoid basidiospores, and the latter has resupinate basidiocarps, larger pores (3–5 per mm) [62], and dextrinoid basidiospores.

Specimen examined: CHINA. Sichuan, Ganluo County, Shengli, Gaoqiao Village, on living tree of *Quercus*, 14 September 2019, Cui 17770 (BJFC).

**Key to species of *Truncatoporia***

1. Basidiocarps resupinate, 3–5 per mm………………………………………………………………………………………………………………………*T. pyricola*
1. Basidiocarps pileate, 6–8 per mm…………………………………………………………………………………………………………………… *T. truncatospora*

***Vanderbyliella*** B.K. Cui & Xing Ji, gen. nov.

MycoBank: MB 847360

Differs from other genera by its pileate basidiocarps with an orange brown pileal surface, a dimitic hyphal system with strongly dextrinoid skeletal hyphae, and hyaline, thick-walled, ellipsoid, non-truncate, and cyanophilous basidiospores.

Type species: *Vanderbyliella tianmuensis* (B.K. Cui & C.L. Zhao) B.K. Cui & Xing Ji

Etymology: Vanderbyliella (Lat.) refers to the morphological similarity to *Vanderbylia*.

Basidiocarps are annual to perennial, pileate, and hard corky to woody hard when dry. Pileal surface is clay-buff, orange-brown to yellowish brown, glabrous, and concentrically sulcate. Pore surface is buff to pale brown when dry; pores are round to angular; dissepiments thin, entire. Context cream to pale brown, corky to hard corky. Tubes are buff to pale brown, hard corky to woody hard. Hyphal system is dimitic; generative hyphae with clamp connections; skeletal hyphae are strongly dextrinoid and CB+; tissues are unchanged in KOH. Cystidia are absent; cystidioles are present. Basidiospores are ellipsoid, non-truncate, hyaline, thick-walled, smooth, dextrinoid or not, and CB+.

Notes: In our phylogenetic studies, *Perenniporia tianmuensis* B.K. Cui & C.L. Zhao and an unknown species, grouped together and formed a strongly supported clade (99% BS, 1.00 BPP, Figure 1; 100% BS, 1.00 BPP, Figure 2), which was distant from the *Perenniporia* s. s. clade. Morphologically, these two species have pileate basidiocarps and non-truncate basidiospores, which are different from species of *Perenniporia* s. s., so *Vanderbyliella* gen. nov. is proposed.

***Vanderbyliella tianmuensis*** (B.K. Cui & C.L. Zhao) B.K. Cui & Xing Ji, comb. nov.

MycoBank: MB 847394

Basionym: *Perenniporia tianmuensis* B.K. Cui & C.L. Zhao, Mycoscience 54: 236 (2013).

For a detailed description of *Perenniporia tianmuensis*, see Zhao and Cui [18].

Notes: *Vanderbyliella tianmuensis* was first described in *Perenniporia* from China by Zhao and Cui [18]; it is characterized by annual and pileate basidiocarps, a dimitic hyphal system with strongly dextrinoid skeletal hyphae, and thick-walled, ellipsoid, non-truncate, dextrinoid, and cyanophilous basidiospores.

Specimens examined: CHINA. Zhejiang, Lin’an County, Tianmushan Nature Reserve, on the basis of dead angiosperm trees, 10 October 2005, Cui 2648 (holotype, BJFC).

***Xanthoperenniporia*** B.K. Cui & Xing Ji, gen. nov.

MycoBank: MB 847361

Differs from other genera by its resupinate basidiocarps with yellow pore surface, weakly dextrinoid, and cyanophilous skeletal hyphae, hyaline, thick-walled, ellipsoid, truncate, and cyanophilous basidiospores.

Type species: *Xanthoperenniporia tenuis* (Schwein.) B.K. Cui & Xing Ji

Etymology: *Xanthoperenniporia* (Lat.) refers to resembling *Tropicoporia* but with a yellow pore surface.

Basidiocarps are annual to perennial, resupinate to reflexed-effused, and corky when dry. Pore surface is cream to yellow when fresh, buff to yellow; pores are round to angular. Subiculum is thin, cream, buff to pale yellowish brown. Tubes are concolorous with pore surface. Hyphal system is dimitic to trimitic, generative hyphae with clamp connections; skeletal hyphae are dextrinoid or weakly dextrinoid and CB+; tissues are unchanged in KOH. Cystidia are absent, cystidioles are usually present. Basidiospores are ellipsoid, truncate, hyaline, thick-walled, smooth, dextrinoid or not, and CB+.

Notes: In the combined five-gene phylogeny, *Perenniporia maackiae* (Bondartsev & Ljub.) Parmasto, *P. punctata* Hai J. Li & Jing Si, *P. subcorticola* Chao G. Wang & F. Wu and *P. tenuis* (Schwein.) Ryvarden clustered together and formed a single clade with good support (74% BS, 0.96 BPP, Figure 1; 89% BS, 1.00 BPP, Figure 2), which was distant from the *Perenniporia* s. s. clade. Morphologically, these species differ from *Perenniporia* s. s. in having a yellow pore surface. Therefore, the new genus *Xanthoperenniporia* is set up, and four new combinations are proposed.

***Xanthoperenniporia maackiae*** (Bondartsev & Ljub.) B.K. Cui & Xing Ji, comb. nov.

MycoBank: MB 847395

Basionym: *Fomitopsis maackiae* Bondartsev & Ljub., Botanicheskie Materialy 15: 103 (1962).

= *Perenniporia maackiae* (Bondartsev & Ljub.) Parmasto, Ann. Bot. fenn. 32(4): 223 (1995).

For a detailed description of *Perenniporia maackiae*, see Cui et al. [3].

Notes: *Xanthoperenniporia maackiae* grows mainly on *Maackia*. *Xanthoperenniporia subcorticola* is similar to *X. maackiae* by sharing resupinate basidiocarps with yellow pore surfaces, similar sized pores, and the presence of cystidioles, but *X. subcorticola* has smaller basidiospores (4.2–5 × 3.5–4.2 µm) [29], and *X. maackiae* has larger basidiospores (5.4–6.3 × 3.8–5.0 µm).

Specimens examined: CHINA. Heilongjiang, Yichun, Dailing, Liangshui Nature Reserve, on fallen branch of *Maackia*, 26 August 2014, Cui 11531 (BJFC). Jilin, Antu County, Changbaishan Nature Reserve, on dead tree of *Maackia*, 11 September 2014, Dai 14780 (BJFC).

***Xanthoperenniporia punctata*** (Hai J. Li & Jing Si) B.K. Cui & Xing Ji, comb. nov.

MycoBank: MB 847396

Basionym: *Perenniporia punctata* Hai J. Li & Jing Si, Phytotaxa 360(1): 56 (2018).

For a detailed description of *Perenniporia punctata*, see Li et al. [26].

Notes: *Xanthoperenniporia punctata* was recently described in *Perenniporia* from China by Li et al. [26] and is characterized by annual and resupinate basidiocarps with a buff-yellow pore surface, a dimitic hyphal system with slightly dextrinoid skeletal hyphae, broadly ellipsoid to subglobose, and truncate and non-dextrinoid basidiospores.

Specimens examined: CHINA. Hubei, Yichang, Wufeng County, Chaibuxi National Forestry Park, on angiosperm stump, 15 August 2017, Dai 17923 (holotype, BJFC), on rotten wood of *Quercus*, 14 August 2017, Dai 17916 (paratype, BJFC).

***Xanthoperenniporia subcorticola*** (Chao G. Wang & F. Wu) B.K. Cui & Xing Ji, comb. nov.

MycoBank: MB 847397

Basionym: *Perenniporia subcorticola* Chao G. Wang & F. Wu, MycoKeys 69: 62 (2020).

For a detailed description of *Perenniporia subcorticola*, see Wang et al. [29].

Notes: *Xanthoperenniporia subcorticola* was recently described in *Perenniporia* as *P. subcorticola* Chao G. Wang & F. Wu by Wang et al. [29]. It is similar to *Perenniporia corticola* by having a yellow pore surface, dimitic hyphal system, and truncate and dextrinoid basidiospores of almost the same size, but *P. corticola* differs from *P. subcorticola* by having arboriform skeletal hyphae and dendrohyphidia [53].

Specimens examined: CHINA. Fujian, Wuyishan County, Wuyishan Nature Reserve, on rotten wood of *Pinus*, 21 October 2005, Dai 7330 (holotype, BJFC).

***Xanthoperenniporia tenuis*** (Schwein.) B.K. Cui & Xing Ji, comb. nov.

MycoBank: MB 847398

Basionym: *Polyporus tenuis* Schwein., Trans. Am. phil. Soc., New Series 4: 159 (1832).

= *Perenniporia tenuis* (Schwein.) Ryvarden, Norw. J Bot. 20: 9 (1973).

For a detailed description of *Perenniporia tenuis*, see Cui et al. [3].

Notes: *Xanthoperenniporia punctata* is similar to *X. tenuis* in having resupinate basidiocarps, pale yellow to buff-yellow pore surface, and similar sized basidiospores, but the former differs in its smaller pores (6–9 per mm), and the absence of cystidioles and non-dextrinoid basidiospores [26], the latter has larger pores (4–6 per mm), the presence of cystidioles, and dextrinoid basidiospores.

Specimens examined: CHINA. Hubei, Yichang, Wufeng County, Chaibuxi National Forestry Park, on rotten angiosperm stump, 15 August 2017, Dai 17935 (BJFC). Shanxi, Yangcheng County, Manghe Nature Reserve, on fallen trunk of *Vitex*, 25 August 2016, Dai 17026 (BJFC).

**Key to species of *Xanthoperenniporia***

1. Basidiospores non-dextrinoid .……………………………………………………………………………………………………………………………… *X. punctata*
1. Basidiospores dextrinoid …………………………………………………………………………………………………………………………………………………2
2. Basidiocarps resupinate to reflexed-effused, growing on *Maackia*……………………………………………………………………………………… *X. maackiae*
2. Basidiocarps resupinate, growing on other trees………………………………………………………………………………………………………………………3
3. Basidiocarps annual, pores 4–6 per mm ……………………………………………………………………………………………………………………… *X. tenuis*
3. Basidiocarps perennial, pores 7–8 per mm………………………………………………………………………………………………………………*X. subcorticola*

***Yuchengia*** B.K. Cui & K.T. Steffen, Nordic J. Bot. 31(3): 333 (2013).

MycoBank: MB 563490

Type species: *Yuchengia narymica* (Pilát) B.K. Cui, C.L. Zhao & K.T. Steffen, Nordic J. Bot. 31(3): 333 (2013).

Basidiocarps are annual to perennial, resupinate, corky when fresh, and hard corky when dry. Pore surface is cream, yellowish buff to tan; pores are round to angular, dissepiments thin, entire. Subiculum is cream to pale ochraceous. Tubes are concolorous with pore surface. Hyphal system is dimitic; generative hyphae with clamp connections; skeletal hyphae amyloid or not, acyanophilous or weakly cyanophilous, dissolving in KOH. Cystidia are absent; cystidioles are present. Basidiospores are ellipsoid, truncate or not, hyaline, thick-walled, smooth, IKI−, and CB+.

Notes: The type species *Yuchengia narymica* was first described as *Trametes narymica* Pilát [63] and later was transferred to *Perenniporia* by Pouzar [64]. Zhao et al. [35] proposed *Yuchengia* to accommodate *Perenniporia narymica* based on molecular data and morphological characteristics.

***Yuchengia kilemariensis*** (Spirin & Shirokov) B.K. Cui & Xing Ji, comb. nov.

MycoBank: MB 847399

Basionym: *Perenniporia kilemariensis* Spirin & Shirokov, Folia Cryptogamica Petropolitana (Sankt-Peterburg) 6: 38 (2005).

For a detailed description of *Perenniporia kilemariensis*, see Spirin et al. [9].

Notes: *Yuchengia kilemariensis* was originally described from Russia as *Perenniporia kilemariensis* by Spirin et al. [9]. It has inamyloid skeletal hyphae and truncate basidiospores, which is different from the original descriptions of *Yuchengia*. However, it has resupinate basidiocarps, a dimitic hyphal system with skeletal hyphae dissolving in KOH, and ellipsoid and non-dextrinoid basidiospores. These characters fit *Yuchengia* well. Moreover, in ITS + nLSU and five-gene phylogenetic analysis, the sequence of *Y. kilemariensis* from the type specimen fell into *Yuchengia* (Figure 1 and Figure 2). Therefore, *P. kilemariensis* is transferred to *Yuchengia*.

**Key to species of *Yuchengia***

1. Skeletal hyphae amyloid …………………………………………………………………………………………………………………………………… *Y. narymica*
1. Skeletal hyphae inamyloid……………………………………………………………………………………………………………………………… *Y. kilemariensis*

***Microporellus subadustus*** (Z.S. Bi & G.Y. Zheng) B.K. Cui & Xing Ji, comb. nov.

MycoBank: MB 847400

Basionym: *Wrightoporia subadusta* Z.S. Bi & G.Y. Zheng, Bull. Bot. Res., Harbin 7(4): 76 (1987).

= *Perenniporia subadusta* (Z.S. Bi & G.Y. Zheng) Y.C. Dai, Ann. Bot. fenn. 39(3): 180 (2002).

= *Murinicarpus subadustus* (Z.S. Bi & G.Y. Zheng) B.K. Cui & Y.C. Dai, Fungal Di-versity 97: 255 (2019).

= *Perenniporia cystidiata* Y.C. Dai, W.N. Chou & Sheng H. Wu, Mycotaxon 83: 209 (2002).

Notes: After studying type materials of *Wrightoporia subadusta* and *Perennipori cystidiata*, Cui et al. [3] found that the two represent the same species and separated this species from *Perenniporia* and proposed *Murinicarpus*. *Murinicarpus* has the same characters with *Microporellus* Murrill, stipitate basidiocarps, dextrinoid skeletal hyphae, non-truncate and non-dextrinoid basidiospores [33], and it is treated as a synonym of *Microporellus* in the current study.

**Key to species of *Perenniporia* and related genera**

1. Basidiocarps stipitate………………………………………………………………………………………………………………………………………*Microporellus*
1. Basidiocarps resupinate, effused-reflexed to pileate…………………………………………………………………………………………………………………2
2. Basidiospores pale yellowish  …………………………………………………………………………………………………………………………… *Abundisporus*
2. Basidiospores hyaline…………………………………………………………………………………………………………………………………………………… 3
3. Skeletal hyphae amyloid…………………………………………………………………………………………………………………………………………………4
3. Skeletal hyphae inamyloid………………………………………………………………………………………………………………………………………………6
4. Basidiocarps pileate……………………………………………………………………………………………………………………………………………*Minoporus*
4. Basidiocarps resupinate to effused-reflexed ………………………………………………………………………………………………………………………… 5
5. Basidiospores amyloid………………………………………………………………………………………………………………………………………*Amylosporia*
5. Basidiospores inamyloid………………………………………………………………………………………………………………………………………*Yuchengia*
6. Basidiocarps with rhizomorphs .……………………………………………………………………………………………………………………………………… 7
6. Basidiocarps without rhizomorphs…………………………………………………………………………………………………………………………………… 8
7. Pore surface orange to orange-brown  ………………………………………………………………………………………………………………… *Aurantioporia*
7. Pore surface grayish white to pale buff…………………………………………………………………………………………………………… *Rhizoperenniporia*
8. Cystidia present………………………………………………………………………………………………………………………………………………………… 9
8. Cystidia absent………………………………………………………………………………………………………………………………………………………… 10
9. Basidiocarps pileate……………………………………………………………………………………………………………………………………*Hornodermoporus*
9. Basidiocarps resupinate……………………………………………………………………………………………………………………………………*Cystidioporia*
10. Basidiospores non-truncate………………………………………………………………………………………………………………………………………… 11
10. Basidiospores truncate……………………………………………………………………………………………………………………………………………… 15
11. Basidiocarps pileate ………………………………………………………………………………………………………………………………………………… 12
11. Basidiocarps resupinate .…………………………………………………………………………………………………………………………………………… 13
12. Basidiospores obovoid …………………………………………………………………………………………………………………………………… *Vanderbylia*
12. Basidiospores ellipsoid ………………………………………………………………………………………………………………………………… *Vanderbyliella*
13. Basidiocarps annual……………………………………………………………………………………………………………………………………………*Neoporia*
13. Basidiocarps annual to perennial……………………………………………………………………………………………………………………………………14
14. Hyphal system dimitic………………………………………………………………………………………………………………………………*Luteoperenniporia*
14. Hyphal system dimitic to trimitic …………………………………………………………………………………………………………………………… *Poriella*
15. Basidiocarps osseous ………………………………………………………………………………………………………………………………… *Perenniporiopsis*
15. Basidiocarps corky to woody hard…………………………………………………………………………………………………………………………………16
16. Pore surface white when fresh, usually with reddish brown sterile margin…………………………………………………………………………*Niveoporia*
16. Pore surface cream, buff, yellowish to cinnamon, without reddish brown sterile margin………………………………………………………………… 17
17. Basidiocarps resupinate to pileate………………………………………………………………………………………………………………………………… 18
17. Basidiocarps resupinate  …………………………………………………………………………………………………………………………………………… 19
18. Basidiospores > 9 µm in length ………………………………………………………………………………………………………………………… *Truncospora*
18. Basidiospores < 9 µm in length…………………………………………………………………………………………………………………………*Truncatoporia*
19. Tissues brown to black in KOH …………………………………………………………………………………………………………………………………… 20
19. Tissues unchanged in KOH …………………………………………………………………………………………………………………………………………21
20. Dendrohyphidia present at dissepiment edges, basidiospores non-dextrinoid……………………………………………………………………*Dendroporia*
20. Dendrohyphidia usually absent at dissepiment edges, basidiospores dextrinoid…………………………………………………………………*Citrinoporia*
21. Pore surface yellow ……………………………………………………………………………………………………………………………… *Xanthoperenniporia*
21. Pore surface usually cream to cinnamon………………………………………………………………………………………………………………………… 22
22. Basidiospores ≥ 9 µm in length ………………………………………………………………………………………………………………………… *Macrosporia*
22. Basidiospores ≤ 9 µm in length …………………………………………………………………………………………………………………………………… 23
23. Skeletal hyphae usually non-dextrinoid ……………………………………………………………………………………………………………… *Tropicoporia*
23. Skeletal hyphae usually dextrinoid ……………………………………………………………………………………………………………………………… 24
24. Basidiospores < 6 µm in length ………………………………………………………………………………………………………………………… *Perenniporia*
24. Basidiospores > 6 µm in length .………………………………………………………………………………………………………………………… *Macroporia*

## 4. Discussion

The phylogenetic analyses carried out by Binder et al. [45] placed *Perenniporia* in the core polyporoid clade, with species of *Perenniporia* forming more than one lineage. Our phylogenetic analyses confirmed that *Perenniporia* s. l. is polyphyletic and is nested in the core polyporoid clade.

*Abundisporus* Ryvarden is closely related to some *Perenniporia* species. Ryvarden [65] established *Abundisporus* to accommodate the species with pale yellowish and non-dextrinoid basidiospores, previously accepted in *Loweporus* J.E. Wright. Our phylogenetic analyses showed that *Abundisporus* is closely related to *Cystidioporia*, *Macrosporia,* and *Niveoporia*, but distant from the *Perenniporia* s. s. as previous studies [17,19,31]. Morphologically, *Abundisporus* is distinguished from these genera by its more or less pinkish basidiocarps, non-truncate, and pale yellowish basidiospores [66].

*Truncospora*, typified by *T. ochroleuca* (Berk.) Pilát, was established by Pilát [67]. The genus is characterized by pileate basidiocarps, a dimitic hyphal system with clamped generative hyphae, dextrinoid and cyanophilous skeletal hyphae, and hyaline, thick-walled, truncate, ellipsoid, and strongly dextrinoid basidiospores [68,69]. These characters are the same as those of some species in *Perenniporia* s. l. This genus has often been treated as a synonym of *Perenniporia* since its establishment [32,33,34,70]. Decock and Ryvarden [5] considered that *Perenniporia detrita* (Berk.) Ryvarden, *P. ochroleuca* (Berk.) Pilát and *P. ohiensis* (Berk.) Ryvarden formed a morphologically homogeneous group that could be recognized at the genus level. Later phylogeny showed that these three species formed a monophyletic clade that was distinct from *Perenniporia* s. s. [17,19,31]. Morphologically, *Truncospora* mainly differs from *Perenniporia* s. s. by having button-shaped to ungulate basidiocarps, variably dextrinoid skeletal hyphae, and longly ovoid basidiospores [68].

*Vanderbylia* was established by Reid [71]; species in this genus have pileate and hard corky basidiocarps, a dimitic hyphal system with clamped generative hyphae, dextrinoid and cyanophilous skeletal hyphae, and hyaline, thick-walled, non-truncate, subglobose to obovoid, dextrinoid, and cyanophilous basidiospores [3,17]. It was treated as a synonymy of *Perenniporia* [32,72]. Recent phylogenetic analyses revealed that *Vanderbylia* is an independent genus that is distant from *Perenniporia* s. s. [3,17,18,19]. In our ITS + nLSU phylogeny, *Vanderbylia* is closely related to *Vanderbyliella*, but the latter has ellipsoid and dextrinoid or non-dextrinoid basidiospores. *Vanderbylia* differs from *Perenniporia* s. s. by its pileate basidiocarps, dextrinoid skeletal hyphae, and non-truncate and subglobose to amygdaliform basidiospores.

*Hornodermoporus* was established by Teixeira [73] and typified by *H. martius* (Berk.) Teixeira. The genus is characterized by its pileate basidiocarps with a black crust at the pileal surface, a dimitic hyphal system with strongly dextrinoid and cyanophilous skeletal hyphae, the presence of cystidia, truncate, oblong-ellipsoid, and strongly dextrinoid basidiospores. *Hornodermoporus* was treated as a synonym of *Perenniporia* [1,58], but morphologically it differs from *Perenniporia* s. s. in having pileate basidiocarps with a black crusted pileal surface, dextrinoid skeletal hyphae, the presence of cystidia, and oblong-ellipsoid basidiospores. In our phylogeny, the separation of *Hornodermoporus* from *Perenniporia* is supported, as previously reported [3,19,20,36,74].

*Amylosporia*, *Murinicarpus*, *Perenniporiopsis*, *Poriella,* and *Yuchengia* have recently been segregated from *Perenniporia* s. l. These genera were phylogenetically distant from *Perenniporia* s. s. in our study. *Amylosporia* differs from *Perenniporia* s. s. by its amyloid skeletal hyphae and basidiospores [3]. *Amylosporia hattorii* and *Perenniporia amylodextrinoidea* Gilb. & Ryvarden both have amyloid skeletal hyphae, but the former has amyloid and larger basidiospores (10–12 × 5.5–7.5 μm) [3], and the latter has dextrinoid and smaller basidiospores (4.5–5.5 × 3–3.5 μm) [33]. *Murinicarpus*, newly proposed by Cui et al. [3] based on *Wrightoporia subadusta* = *Perenniporia cystidiata*, has same characters with *Microporellus* and is a synonym of *Microporellus*. It is different from *Perenniporia* s. s. by its stipitate basidiocarps, dextrinoid skeletal hyphae, presence of thick-walled cystidia, non-truncate and non-dextrinoid basidiospores [3]. *Perenniporiopsis* is distinguished from *Perenniporia* s. s. by its basidiocarps waxy when fresh and rigidly osseous when dry, dextrinoid skeletal hyphae, and large basidiospores [36]. Chen et al. [37] described *Poriella* as having non-dextrinoid basidiospores. *Perenniporia africana* and *P. ellipsospora* are congeneric, although they have dextrinoid basidiospores; they are transferred to *Poriella*. *Poriella* is distinguished from *Perenniporia* s. s. by its unbranched, dextrinoid skeletal hyphae, and non-truncate basidiospores. *Perenniporia kilemariensis*, which has inamyloid skeletal hyphae and truncate basidiospores is transferred to *Yuchengia*, so the definition of *Yuchengia* is revised also in this study. *Yuchengia* differs from *Perenniporia* s. s. by its non-dextrinoid basidiospores, unbranched “amyloid” skeletal hyphae.

The segregation of *Aurantioporia*, *Citrinoporia*, *Cystidioporia*, *Dendroporia*, *Luteoperenniporia*, *Macroporia*, *Macrosporia*, *Minoporus*, *Neoporia*, *Niveoporia*, *Rhizoperenniporia*, *Tropicoporia*, *Truncatoporia*, *Vanderbyliella*, and *Xanthoperenniporia* from *Perenniporia* s. l. was well supported by phylogenetic analyses of ITS + nLSU and ITS + nLSU + mtSSU + TEF1 + TBB1. In the ITS + nLSU gene phylogenetic tree, *Yuchengia* is closely related to *Poriella*; in the combined five-gene phylogenetic analysis, *Yuchengia* was related to *Minoporus*, *Neoporia*, *Poriella*, and *Vanderbyliella*. However, *Yuchengia* has resupinate basidiocarps, amyloid or not, acyanophilous or weakly cyanophilous skeletal hyphae, and non-dextrinoid basidiospores; *Minoporus* produces pileate basidiocarps, cyanophilous skeletal hyphae, and truncate and dextrinoid basidiospores; *Neoporia* has dextrinoid and cyanophilous skeletal hyphae and dextrinoid basidiospores; *Poriella* has dextrinoid and cyanophilous skeletal hyphae [37]; *Vanderbyliella* has pileate basidiocarps and dextrinoid and cyanophilous skeletal hyphae.

According to phylogenetic analyses and morphological characters, some species of *Perenniporia* s. l. have a new generic placement. For four species in the phylogenetic tree, including *Perenniporia tephropora* (Mont.) Ryvarden, *P. subtephropora* B.K. Cui & C.L. Zhao, *P. eugeissonae* P. Du & Chao G. Wang and *P. straminea* (Bres.) Ryvarden, due to unclear phylogenetic relationships and insufficient morphological difference from *Perenniporia* s. s. species, their taxonomic positions were remained in *Perenniporia* s. l.

In summary, we carried out a comprehensive study on *Perenniporia* s. l., 44 species of *Perenniporia* s. l. with available sequences. According to phylogenetic evidence and morphological characteristics, 15 new genera were set up, 2 new species were described, and 37 new combinations were proposed. Species lacking reliable molecular sequences were not included in this study.

## Figures and Tables

**Table 1:** Samples and GenBank accession numbers of sequences used in this study.

Species	Sample no.	Locality	GenBank Accession No.				
			ITS	nLSU	mtSSU	TEF1	TBB1
*Abortiporus biennis*	FD-319	the United States of America	KP135300	KP135195	—	—	—
*Abundisporus pubertatis*	Dai 11927	China	KC787569	KC787576	KF051034	KF181128	KF482828
*Abundisporus sclerosetosus*	MUCL 41438	Singapore	FJ411101	FJ393868	—	—	—
*Abundisporus violaceus*	Ryvarden 32807	Zimbabwe	KF018127	KF018135	KF051038	KF181132	KF482832
*Amylosporia hattorii*	Cui 10912	China	KX900675	KX900725	—	KX900852	—
*Amylosporia hattorii*	Dai 10315	China	JQ861740	JQ861756	KF218290	—	—
*Antrodia albida*	FP-105979	Unknown	EU232189	EU232272	—	—	—
*Antrodia serialis*	FP-133692	the United States of America	KC585303	KC585127	—	—	—
*Aurantioporia aurantiaca*	CBS 125867	French Guiana	MH863779	MH875242	—	—	—
*Aurantioporia bambusicola*	Cui 11050	China	KX900668	KX900719	KX900771	**OK665240**	—
*Aurantioporia bambusicola*	Yuan 3925	China	JQ861736	—	—	—	—
*Bjerkandera adusta*	HHB-12826-Sp	the United States of America	KP134983	KP135198	—	—	—
*Cerrena unicolor*	FD-299	the United States of America	KP135304	KP135209	—	—	—
*Citrinoporia citrinoalba*	Cui 13615	China	MG847215	MG847224	MG847238	MG867708	—
*Citrinoporia citrinoalba*	Dai 13643	China	KX880622	KX880661	KX880705	—	—
*Citrinoporia corticola*	Dai 18633	Malaysia	MT117217	MT117222	**OK642017**	**OK665241**	—
*Citrinoporia corticola*	Dai 18641	Malaysia	MT117218	MT117223	—	—	—
*Climacocystis borealis*	FD-31	the United States of America	KP135308	KP135210	—	—	—
*Coriolopsis dendriformis*	Cui 6719	China	KC867408	KC867445	—	—	—
*Coriolopsis retropicta*	Cui 13849	China	MK116481	MK116490	—	—	—
*Crassisporus imbricatus*	Dai 10788	China	KC867350	KC867425	—	—	—
*Crassisporus macroporus*	Cui 14468	China	MK116486	MK116495	—	—	—
*Cryptoporus sinensis*	Cui 17418	China	**OK642180**	**OK642231**	—	—	—
*Cryptoporus volvatus*	CBS 432.48	Canada	MH856424	MH867970	—	—	—
*Cystidioporia piceicola*	Cui 10460	China	JQ861742	JQ861758	KF218310	KF286316	KF482803
*Cystidioporia piceicola*	Dai 4181	China	JF706328	JF706336	KF218311	KF286317	KF482802
*Daedalea quercina*	Dai 12659	Finland	KP171208	KP171230	KR605990	KR610719	
*Dendroporia cinereofusca*	Cui 5280	China	KF568892	KF568894	—	—	—
*Dendroporia cinereofusca*	Dai 9289	China	KF568893	KF568895	—	—	—
*Dichomitus squalens*	Cui 9639	China	JQ780407	JQ780426	—	—	—
*Dichomitus squalens*	CBS 432.34	Poland	MH855594	MH867104	—	—	—
*Donkioporia expansa*	MUCL 35116	Belgium	FJ411104	FJ393872	—	—	—
*Earliella scabrosa*	PR1209	Puerto Rico	JN165009	JN164793	—	—	—
*Fomitopsis pinicola*	Cui 10405	China	KC844852	KC844857	KR605961	KR610690	—
*Fragiliporia fragilis*	Dai 13080	China	KJ734260	KJ734264	—	—	—
*Fragiliporia fragilis*	Dai 13559	China	KJ734261	KJ734265	—	—	—
*Gelatoporia subvermispora*	BRNU 592909	Czech Republic	FJ496694	FJ496706	—	—	—
*Grammothele lineata*	Cui 6539	China	KX832049	KX832058	—	—	—
*Grammothelopsis subtropica*	Cui 9035	China	JQ845094	JQ845097	—	—	—
*Grammothelopsis subtropica*	Cui 9041	China	JQ845096	JQ845099	—	—	—
*Haploporus odorus*	Dai 11296	China	KU941845	KU941869	—	—	—
*Haploporus tuberculosus*	15559	Sweden	KU941857	KU941881	—	—	—
*Heterobasidion annosum*	VL296	Lithuania	JF440572	—	—	—	—
*Hornodermoporus latissimus*	Dai 12054	China	KX900639	KX900686	KF218297	KF286303	KF482789
*Hornodermoporus martius*	Cui 7992	China	HQ876603	HQ654114	KF051041	KF181135	KF482835
*Irpex lacteus*	FD-9	the United States of America	KP135026	KP135224	—	—	—
*Jorgewrightia guangdongensis*	Cui 9130	China	JQ314373	JQ780428	—	—	—
*Jorgewrightia tropica*	Cui 13740	China	KY449438	KY449449	—	—	—
*Lentinus crinitus*	DSH92N43C	Costa Rica	KP283495	KP283523	—	—	—
*Luteoperenniporia australiensis*	Cui 16742	Australia	**OK642220**	**OK642275**	**OK642018**	**OK665242**	—
*Luteoperenniporia australiensis*	Cui 16743	Australia	**OK642221**	**OK642276**	**OK642019**	**OK665243**	**OK665272**
*Luteoperenniporia bannaensis*	Cui 8560	China	JQ291727	JQ291729	KF218280	KF286286	KF482772
*Luteoperenniporia bannaensis*	Cui 8562	China	JQ291728	JQ291730	KF218281	KF286287	KF482773
*Luteoperenniporia mopanshanensis*	CLZhao 2404	China	MH784911	MH784915	—	—	—
*Luteoperenniporia mopanshanensis*	CLZhao 5145	China	MH784912	MH784916	—	—	—
*Luteoperenniporia yinggelingensis*	Cui 13625	China	MH427960	MH427967	MH427975	MH427996	—
*Luteoperenniporia yinggelingensis*	Cui 13627	China	MH427961	MH427968	MH427976	MH427997	—
*Macroporia lacerata*	Cui 7220	China	JX141448	JX141458	KF218295	KF286301	KF482787
*Macroporia lacerata*	Dai11268	China	JX141449	JX141459	KF218296	KF286302	KF482788
*Macroporia macropora*	Zhou 280	China	JQ861748	JQ861764	KF494992	KF482765	KF482798
*Macroporia macropora*	Zhou 407	China	JQ861746	JQ861762	KF494991	KF482767	KF482784
*Macroporia subrhizomorpha*	LWZ 20190722-36	China	MZ578440	MZ578444	—	—	—
*Macroporia subrhizomorpha*	LWZ 20190723-10	China	MZ578441	—	—	—	—
*Macroporia tibetica*	Cui 9457	China	JF706326	JF706332	KF218332	KF286297	KF482782
*Macroporia tibetica*	Cui 9459	China	JF706327	JF706333	KF218333	KF286296	KF482783
*Macrosporia nanlingensis*	Cui 7541	China	HQ848479	HQ848488	KF218306	KF286312	—
*Macrosporia nanlingensis*	Cui 7620	China	HQ848477	HQ848486	KF218307	KF286313	—
*Microporellus subadustus*	Cui 8459	China	HQ876606	HQ654113	—	—	—
*Microporellus subadustus*	Dai 13895	China	KX880621	KX880660	—	KX880879	KX880780
*Minoporus minor*	Dai 9198	China	KF495005	KF495016	KF494994	—	KF494969
*Minoporus minor*	Cui 5782	China	HQ883475	—	KF218300	—	KF494968
*Neoporia bostonensis*	CLZhao 144	the United States of America	MG491283	MG491286	—	—	—
*Neoporia bostonensis*	CLZhao 2854	the United States of America	MG491284	MG491287	—	—	—
*Neoporia koreana*	KUC20080517-02	Republic of Korea	KJ156308	KJ156300	—	—	—
*Neoporia koreana*	KUC20080517-15	Republic of Korea	KJ156309	KJ156301	—	—	—
*Neoporia rhizomorpha*	Dai 7248	China	JF706330	JF706348	KF218315	KF286321	KF482807
*Neoporia rhizomorpha*	Cui 7507	China	HQ654107	HQ654117	KF218314	KF286320	KF482806
*Niveoporia decurrata*	Dai 16637	Thailand	KY475566	**OP289291**	—	**OP296862**	—
*Niveoporia decurrata*	Dai 16660	Thailand	KY475567	**OP289292**	—	**OP296863**	—
*Niveoporia russeimarginata*	Yuan 1244	China	JQ861750	JQ861766	KF218316	KF286322	KF482808
*Niveoporia russeimarginata*	Yuan 1262	China	JQ861751	JQ861767	KF218317	KF286323	KF482809
*Niveoporia subrusseimarginata*	Cui 16980	China	**OK642223**	**OK642278**	—	**OK665245**	—
*Niveoporia subrusseimarginata*	Cui 16991	China	**OK642224**	**OK642279**	—	**OK665246**	**OK665273**
*Obba valdiviana*	Gates FF503	Australia	HQ659235	HQ659235	—	—	—
*Perenniporia eugeissonae*	Dai 18600	Malaysia	MT232518	MT232512	—	—	—
*Perenniporia eugeissonae*	Dai 18605	Malaysia	MT232519	MT232513	—	—	—
*Perenniporia hainaniana*	Cui 6364	China	JQ861743	JQ861759	KF051044	KF181138	KF482838
*Perenniporia hainaniana*	Cui 6365	China	JQ861744	JQ861760	KF051045	KF181139	KF482839
*Perenniporia medulla-panis*	Cui 14515	China	MG847214	MG847223	—	MG867707	MG867711
*Perenniporia medulla-panis*	MUCL 43250	Norway	FJ411087	FJ393875	—	—	—
*Perenniporia straminea*	Cui 8718	China	HQ876600	JF706335	KF218318	KF286324	KF482810
*Perenniporia straminea*	Cui 8858	China	HQ654104	JF706334	KF218319	KF286325	KF482811
*Perenniporia substraminea*	Cui 10177	China	JQ001852	JQ001844	KF051046	KF181140	KF482840
*Perenniporia substraminea*	Cui 10191	China	JQ001853	JQ001845	KF051047	KF181141	KF482841
*Perenniporia subtephropora*	Dai 10962	China	JQ861752	JQ861768	KF218323	KF286329	KF482815
*Perenniporia subtephropora*	Dai 10964	China	JQ861753	JQ861769	KF218324	KF286330	KF482816
*Perenniporia tephropora*	Cui 8040	China	JN048763	HQ654118	KF218328	KF286307	KF482793
*Perenniporia tephropora*	Cui 9029	China	HQ876601	JF706339	KF218327	KF286306	KF482792
*Perenniporiella chaquenia*	MUCL 47647	Argentina	FJ411083	FJ393855	—	HM467609	—
*Perenniporiella micropora*	MUCL 43581	Cuba	FJ411086	FJ393858	—	HM467608	—
*Perenniporiella pendula*	MUCL 46034	Cuba	FJ411081	FJ393853	—	HM467601	—
*Perenniporiopsis minutissima*	Dai 12457	China	KF495004	KF495014	KF218302	KF286308	KF482794
*Perenniporiopsis minutissima*	Cui 10979	China	KF495003	KF495013	KF218304	KF286310	KF482796
*Phanerochaete carnosa*	HHB-9195-Sp	the United States of America	KP135129	KP135242	—	—	—
*Phlebia radiata*	AFTOL-484	Unknown	AY854087	AF287885	—	—	—
*Piloporia sajanensis*	HK 17250 (GB)	Russia	JX109853	JX109853	—	—	—
*Polyporus tuberaster*	Dai 12462	China	KU507580	KU507582	—	—	—
*Poriella africana*	Cui 8674	China	KF018119	KF018128	KF218276	KF286282	KF482768
*Poriella africana*	Cui 8676	China	KF018120	KF018129	KF218277	KF286283	KF482769
*Poriella ellipsospora*	Cui 10276	China	KF018124	KF018132	KF218286	KF286292	KF482778
*Poriella ellipsospora*	Cui 10284	China	JQ861739	KF018133	KF218285	KF286291	KF482777
*Poriella subacida*	Cui 10053	China	KF495006	KF495017	KF218321	KF286327	KF482813
*Poriella subacida*	DLL2010-125	the United States of America	JQ673016	—	—	—	—
*Poriella valliculorum*	LE 222974	Russia	KM411458	KM411474	—	KM411489	—
*Pyrofomes demidoffii*	PRM869997	Macedonia	KY940246	KY940259	—	—	—
*Rhizoperenniporia japonica*	Cui 7047	China	KX900677	KX900727	KF218294	KF286300	KF482786
*Rhizoperenniporia japonica*	Cui 9181	China	JQ001856	JX141468	KF218293	KF286299	—
*Sebipora aquosa*	Miettinen 8680.1	Indonesia	HQ659240	HQ659240	—	—	—
*Skeletocutis amorpha*	Miettinen 11038.1	Finland	FN907913	FN907913	—	—	—
*Sparsitubus nelumbiformis*	Cui 8497	China	KX880631	KX880670	KX880714	KX880887	KX880786
*Sparsitubus nelumbiformis*	Cui 6590	China	KX880632	KX880671	KX880715	KX880888	—
*Steccherinum laeticolor*	FP-102480-sp	the United States of America	KY948823	KY948868	—	—	—
*Stereum hirsutum*	NBRC 6520	Unknown	AY854063	AF393078	—	—	—
*Trametes elegans*	FP-105679-Sp	the United States of America	JN164944	JN164799	—	—	—
*Trametes hirsuta*	RLG-5133-T	the United States of America	JN164941	JN164801	—	—	—
*Trametes polyzona*	BKW004	Ghana	JN164978	JN164790	—	—	—
*Trametes suaveolens*	FP-102529-Sp	the United States of America	JN164966	JN164807	—	—	—
*Trametes versicolor*	FP-135156-Sp	the United States of America	JN164919	JN164809	—	—	—
*Tropicoporia aridula*	Dai 12396	China	JQ001854	JQ001846	KF218278	KF181158	—
*Tropicoporia aridula*	Dai 12398	China	JQ001855	JQ001847	KF218279	KF286285	KF482771
*Tropicoporia brasiliensis*	AN-MA10	Brazil	KX584437	KX619594	—	—	—
*Tropicoporia brasiliensis*	URM89948	Brazil	KX584436	—	—	—	—
*Tropicoporia centrali-africana*	URM 82624	Brazil	KX584433	KX619599	—	—	—
*Tropicoporia centrali-africana*	URM 82568	Brazil	KX584432	KX619598	—	—	—
*Tropicoporia vanhulleae*	MUCL 46315	Senegal	KP217810	—	—	—	—
*Tropicoporia vanhulleae*	MUCL 38450	Zimbabwe	KP217811	—	—	—	—
*Truncatoporia pyricola*	Cui 9149	China	JN048762	JN048782	—	KF286318	KF482804
*Truncatoporia pyricola*	Dai 10265	China	JN048761	JN048781	—	KF286319	KF482805
*Truncatoporia truncatospora*	Cui 6987	China	JN048778	HQ654112	KF218334	KF286288	KF482774
*Truncatoporia truncatospora*	Dai 5125	China	HQ654098	HQ848481	KF218335	KX880880	KF482770
*Truncospora macrospora*	Cui 8106	China	JX941573	JX941596	KX880763	KX880920	KX880809
*Truncospora ochroleuca*	Cui 16795	Australia	**OK642218**	**OK642273**	**OK642015**	**OK665238**	—
*Truncospora ochroleuca*	JV0610/7B	Belize	KJ410698	—	—	KJ410718	—
*Truncospora ohiensis*	MUCL 41036	the United States of America	FJ411096	FJ393863	—	—	—
*Truncospora ornata*	SP6672	Russia	KJ410690	—	—	KJ410712	—
*Tyromyces chioneus*	FD-4	the United States of America	KP135311	KP135291	—	—	—
*Vanderbylia cinnamomea*	CLZhao 8952	China	MT372778	MT372788	—	—	—
*Vanderbylia delavayi*	Dai 6891	China	JQ861738	—	KF218287	KF286293	KF482779
*Vanderbylia fraxinea*	Cui 8871	China	JF706329	JF706345	KF051050	KF181144	KF482844
*Vanderbylia vicina*	MUCL 44779	Ethiopia	FJ411095	FJ393862	—	—	—
*Vanderbyliella* sp.	Knudsen 04-111	China	JQ861737	JQ861755	KF218282	—	KF494963
*Vanderbyliella tianmuensis*	Cui 2648	China	JX141453	JX141463	KF218329	—	KF494971
*Vanderbyliella tianmuensis*	Cui 2715	China	JX141454	JX141464	KF218331	—	KF494972
*Xanthoperenniporia maackiae*	Dai 8929	China	HQ654102	JF706338	KF218299	KF286305	KF482791
*Xanthoperenniporia maackiae*	Cui 10092	China	KX900680	KX900730	KX900780	KX900856	—
*Xanthoperenniporia punctata*	Dai 17916	China	MG869686	MG869688	—	—	—
*Xanthoperenniporia punctata*	Dai 17923	China	MG869687	MG869689	**OP293085**	**OP296864**	—
*Xanthoperenniporia subcorticola*	Cui 1248	China	HQ848472	HQ848482	KF218284	KF286290	KF482776
*Xanthoperenniporia subcorticola*	Dai 7330	China	HQ654094	HQ654108	KF218283	KF286289	KF482775
*Xanthoperenniporia tenuis*	Wei 2783	China	JQ001858	JQ001848	KF218325	KF286331	KF482817
*Xanthoperenniporia tenuis*	Wei 2969	China	JQ001859	JQ001849	KF218326	KF286332	KF482818
*Yuchengia kilemariensis*	LE 214743	Russia	KM411457	KM411473	—	KM411488	—
*Yuchengia narymica*	Dai 7050	China	JN048776	JN048795	KF051053	KF181147	KF482836
*Yuchengia narymica*	Dai 10510	China	HQ654101	JF706346	KF051054	KF181148	KF482833

New sequences are shown in bold.

## Data Availability

Publicly available datasets were analyzed in this study. These data can be found here: https://www.ncbi.nlm.nih.gov/genbank/, accessed on 24 August 2022; http://purl.org/phylo/treebase, accessed on 29 November 2022; http://www.mycobank.org/, accessed on 24 January 2023.
